# Brewer’s Spent Yeast as a Biosorbent for the Synthetic Dye Tartrazine Yellow

**DOI:** 10.1021/acsomega.5c05650

**Published:** 2026-02-04

**Authors:** Louise N. N. Lourenço, Ivaldo Itabaiana, Ailton C. Lemes

**Affiliations:** Department of Biochemical Engineering, School of Chemistry, Federal University of Rio de Janeiro, Rio de Janeiro 21941-909, Brazil

## Abstract

Tartrazine is a synthetic dye commonly used in the food industry to enhance the visual appeal of food products. However, its instability under specific conditions, such as changes in pH, exposure to UV or sunlight, or increased temperature, may lead to adverse effects, raising concerns about its toxicity. Thus, ensuring the safety, controlled release, and stability of these colorants in food matrices remains a significant challenge. This study aimed to evaluate inactivated brewer’s yeast (*Saccharomyces cerevisiae*) as a promising biosorbent matrix for the adsorption and stabilization of tartrazine, thereby developing a safer, more stable delivery system for this food additive. Unlike previous studies that focus primarily on wastewater treatment, this work uniquely investigates tartrazine–yeast interactions under food-relevant and simulated gastrointestinal conditions, highlighting the yeast’s ability to stabilize the dye and control its release. Adsorption experiments were conducted at different pH levels (2 and 7) and temperatures (10, 25, 37, and 90 °C). Samples of the dye alone, the yeast alone, and the dye adsorbed onto the yeast were analyzed by Fourier transform infrared spectroscopy (FTIR), thermogravimetric analysis (TGA), and scanning electron microscopy (SEM). The system comprising the yeast with the highest adsorption percentage was investigated for its stability at various pH and temperature conditions, as well as simulated gastrointestinal degradation. The highest adsorption was observed at pH 2 and 25 °C (4.23 mg·g^–1^). The kinetic data fit a pseudo-second-order model, suggesting that chemisorption is driven by electron-sharing or valence interactions between the dye and the yeast surface. FTIR analysis revealed characteristic bands of Brewer’s spent yeast related to hydroxyl groups (around 3271 cm^–1^), C–H stretching vibrations (1398 and 2916 cm^–1^), carbonyl groups (1633 cm^–1^), and aromatic residues (between 669 and 536 cm^–1^). No significant disappearance of S=O bands was observed after adsorption. Still, shifts and the appearance of peaks indicate chemical interactions between dye molecules and yeast cell wall components under different pH conditions. TGA results showed an increase in the thermal stability of the adsorbed dye, with lower mass loss than free tartrazine. Isotherm modeling revealed that the Temkin model best described adsorption at pH 2, indicating a decreasing interaction energy with increasing surface coverage, whereas the Dubinin–Radushkevich model provided the best fit at pH 7, suggesting a physical adsorption mechanism on a porous biosorbent surface. Simulated gastrointestinal conditions revealed lower dye desorption (2.37 mg·g^–1^) from the biosorbent at pH 7 and 37 °C, indicating potential for controlled release. This study aims to demonstrate a novel role for residual beer yeast as a stabilizing matrix and controlled-release system for tartrazine under simulated gastrointestinal conditions. It highlights the importance of brewer’s yeast as a sustainable, functional, and promising biosorbent for the formulation of future food compounds, mitigating the adverse effects and toxicity associated with free tartrazine and thereby contributing to safer applications of food additives.

## Graphical abstract

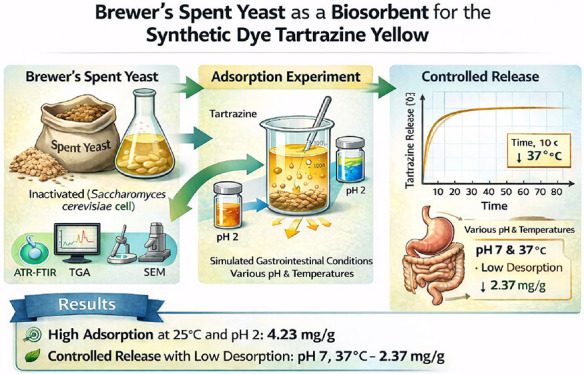

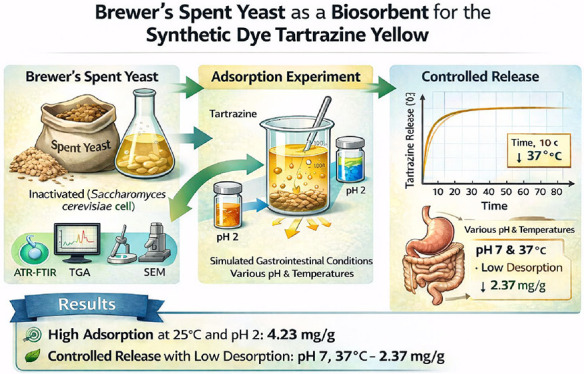

## 1 Introduction

Color strongly influences the popularity, acceptance, and preference of foods and beverages. Therefore, the use of colorants is essential to generate color attractiveness and enhance the product’s appeal to consumers.^1^ Among these, azo dyes represent a significant class of synthetic colorants widely used in the food industry. These compounds are characterized by the presence of an azo group (−N=N−) linked to aromatic structures, which generate intense and stable colors.^2^ However, under certain operational, chemical, or enzymatic conditions (enzymatic reduction, interaction with food matrices), the azo bond can be cleaved, leading to the formation of aromatic amine compounds recognized for their toxicity, even at low concentrations, and their potential allergenic and carcinogenic effects.^3,4^

One of the most commonly used azo dyes is tartrazine yellow, applied extensively in the food, beverage, textile, cosmetics, and pharmaceutical industries. Although regulatory agencies have established an acceptable daily intake of 0–10 mg/kg of body weight,^4^ growing health concerns regarding its degradation products have driven the search for safer alternatives or stabilization strategies.^5^ In this context, it is necessary to outline approaches to stabilize such compounds, as the industry and consumers prepare to reduce or eliminate the use of synthetic colorants, or as natural alternatives are identified and developed. Preventing the formation or release of toxic aromatic amines (acute and chronic, including genotoxic, mutagenic, and carcinogenic potential), during processing or digestion, is particularly relevant to ensure consumer safety.^5^

Adsorption has emerged as a promising strategy for dye stabilization and removal due to its simplicity, low cost, high efficiency, and retention of the chemical structure of the target compound.^6,11^ Unlike degradation-based treatments, adsorption allows the dye to remain chemically intact, which is crucial in food systems where the functionality and color of additives must be preserved.^7,12^ This process can help the dye maintain its color in the product while increasing its stability against degradation. However, it may also prevent metabolism, potentially leading to undesirable effects on human health. This makes adsorption a rational choice for addressing the challenges associated with tartrazine stabilization under variable environmental conditions.

It is necessary to explore alternative materials that promote dye adsorption, and agro-industrial byproducts have recently attracted attention for this purpose. Such materials offer the opportunity to valorize residues that would otherwise be discarded and cause environmental problems, while simultaneously stabilizing compounds that are potentially harmful to humans but are still used due to the scarcity of stable, natural colorants. Brewer’s spent yeast (BSY), the residual biomass of *Saccharomyces cerevisiae* generated in large quantities during beer production (1.5–3 kg per 100 L of beer, totaling approximately 400 million kg annually),^8^ has recently garnered attention as a sustainable biosorbent. Traditionally used as animal feed, BSY possesses a cell wall rich in β-glucans, mannoproteins, and chitin, offering a wide array of functional groups (e.g., hydroxyl, amine, phosphate) that enable strong interactions with dyes and other molecules^9,10^ Several studies have demonstrated the high biosorption capacity of inactivated yeast for different synthetic dyes. For example, adsorption capacities exceeding 100 mg/g have been reported for Brilliant Red HE-3B.^11^ Rusu et al.^12^ also confirmed the effective adsorption of food dyes such as tartrazine, ponceau 4R, and patent blue without inducing chemical degradation, highlighting the potential of BSY as a safe, food-compatible adsorbent. Moreover, BSY has been successfully incorporated into composite matrices to enhance its reusability and performance for both organic and inorganic contaminants.^13^

Even with these advances, most of the available studies have focused on the use of BSY for dye removal from wastewater or contaminated effluents, as in the work of Shi et al.^2^ and Hussain et al.,^14^ rather than exploring its potential as a biosorbent to stabilize harmful compounds with possible applications in food and beverage systems. There is also a significant gap in understanding how these biosorbents interact with dyes, as evident in the work of Castro et al.,^15^ which evaluates the interaction between anionic dyes, such as tartrazine, by assessing dye bisorption in brewer’s yeast residues. How the resulting complexes resist food-processing conditions and how they behave during gastrointestinal transit. Research on how dye–yeast complexes behave under pH and temperature variations that mimic digestive processes is also interesting, including their potential to control the release or stabilization of colorants during digestion. Addressing these aspects could open new opportunities to develop safer, more functional food formulations while promoting the valorization of industrial byproducts.

There is a limited number of studies evaluating the use of BSY as a biosorbent, and there is a lack of data on the stabilization of dyes by BSY for food applications, as well as on how operational conditions and use in different food matrices can affect the stability of the dye once stabilized in the biomass. This study addresses this critical gap by evaluating BSY not only as a biosorbent but also as a functional delivery system capable of stabilizing tartrazine and controlling its release in gastrointestinal simulation. The novelty of this work lies in providing a sustainable strategy to add value to industrial byproducts, thereby improving the safety and stability of synthetic food dyes. Furthermore, it proposes a sustainable approach to valorize abundant and low-cost industrial waste, supporting the principles of the circular economy and contributing to safer food systems.

## 2 Materials and Methods

### 2.1 Materials

The BSY, *S. cerevisiae*, was kindly provided by a company located in the Technology Park of the Federal University of Rio de Janeiro—RJ (latitude −22.866, longitude −43.216). The BSY predominantly comprises 45.32% protein, 9.90% carbohydrates, 3.10% lipids, 30.2% fiber, 5.58% moisture, and other components in smaller proportions. The material was standardized to a particle size of 32 mesh (Tyler sieve, diameter <0.5 mm) and stored in polyethylene packaging, protected from light, at room temperature (25 °C). The food-grade yellow tartrazine was purchased from NutyLac Food Industry (Sorocaba, São Paulo, Brazil). All other reagents used were of analytical grade and were intended for laboratory use.

### 2.2 Methods

#### 2.2.1 Characterization of the Isolated Synthetic Dye

##### 2.2.1.1 Solubility

The solubility of tartrazine yellow dye was evaluated by dissolving different masses (0.5–10.5 mg) at various pH values (2–8) using McIlvaine buffer at room temperature (25 °C). The dye concentration was determined by UV–vis spectroscopy using a model V-M5-BEL spectrometer, with analysis performed at 425 nm. Standard curves were prepared at different pH values to serve as a basis for subsequent analyses in the study, including pH and point of zero charge (PZC) testing. In addition, pH 2 and pH 7 were selected as the basis for all analyses at different pH values because they represent interesting extremes for comparing the physical–chemical behavior of both the dye and the adsorbent–adsorbate interaction.^16^

##### 2.2.1.2 Influence of pH and Temperature on Dye Stability in Solution

The stability of tartrazine dye was evaluated under different conditions. The dye solutions, at a concentration of 10 mg·L^–1^ in McIlvaine buffer, were incubated at temperatures of 10, 25, 37, and 90 °C, and at pH levels of 2 and 7. The samples were monitored for 96 h to assess possible degradation or changes in absorbance. The concentration was determined by UV–vis spectrophotometry using a V-M5 model by BEL at 425 nm, and the results were expressed as the equilibrium concentration (*C*_e_) in milligrams per liter (mg·L^–1^).

#### 2.2.2 Adsorption of the Yellow Dye Tartrazine in BSY

In this study, the adsorbent dosage was fixed at 0.1 g for all experiments. Preliminary tests indicated that this amount ensured sufficient contact surface while maintaining measurable dye concentrations in solution. Although systematic dosage optimization was not performed, this fixed amount was selected to allow consistent comparison across different parameters.

##### 2.2.2.1 Impact of pH on Dye Adsorption

To determine the effect of pH on dye adsorption onto BSY, solutions containing 10 mg·L^–1^ of dye and 0.1 g of BSY were prepared at different pH values (2 and 7). The prepared solutions were kept under stirring at 200 rpm for 48 h and then centrifuged (5000 rpm, 10 min). The supernatant was read on a UV–vis spectrophotometer (model V-M5, BEL) at 425 nm to determine the residual dye concentration in the solution. The adsorption capacity was calculated using eq 1.

$$q_{e}=\frac{(C_{0}-C_{e})\cdot V}{m}$$ 1

where *Q*_e_ is the adsorption capacity at equilibrium (mg·g^–1^); *C*_0_ is the initial concentration of the dye (mg·L^–1^); *C*_e_ is the concentration of the dye at equilibrium (mg·L^–1^); *V* is the volume of the solution (L); *m* is the mass of the yeast used (g).

The same methodology was used to determine the PZC, comparing the pH values before and after 24 h.^17^

##### 2.2.2.2 Adsorption Kinetics and Isotherm Models

For the kinetic tests, 50 mL solutions of McIlvaine buffer at pHs 2 and 7, to which 10 mg of tartrazine and 0.1 g of previously dried BSY (24 h at 105 °C) were added. The solutions were stirred continuously at room temperature (25 °C), and aliquots of 3 mL were collected at times between 5 and 120 min. After centrifugation (5000*g*, 10 min), the samples were analyzed by UV–vis spectrophotometry (model V-M5—BEL) at 425 nm. The amount of dye adsorbed over time was determined using eq 2. The values of *q_t_* and *C*_e_ were applied to the pseudo-first and pseudo-second-order models, using linear regression to fit and evaluate the coefficient of determination (*R* ^2^).

$$q_{t}=\frac{(C_{0}-C_{t}).V}{m}$$ 2

where *q_t_* is the amount adsorbed at time *t* (mg·g^–1^); *C*_0_ is the initial concentration of the dye (mg·L^–1^); *C_t_* is the concentration at time (mg·L^–1^); *V* is the volume of the solution (L); and *m* is the mass of the yeast used (g).

For the isotherm tests, solutions containing 0.1 g of dry biomass and different concentrations of tartrazine (2.5 to 50 mg·L^–1^) were prepared in triplicate (50 mL each), adjusted to pHs 2 and 7, and kept at 10, 25, 37, and 90 °C. After centrifugation (5000*g*, 10 min), the samples were analyzed by UV–vis spectrophotometry at 425 nm to determine *Q*_e_ and *C*_e_ using eq 3. The data obtained were fitted to isothermal models to describe the adsorption behavior at equilibrium.

$$q_{e}=\frac{(C_{0}-C_{e})\cdot V}{m}$$ 3

where *Q*_e_ is the adsorption capacity at equilibrium (mg·g^–1^); *C*_0_ is the initial concentration of the dye (mg·L^–1^); *C*_e_ is the concentration of the dye at equilibrium (mg·L^–1^); *V* is the volume of the solution (L); and *m* is the mass of the yeast used (g).

##### 2.2.2.3 Thermodynamic Parameters

Adsorption experiments were carried out at four temperatures (10, 25, 37, and 90 °C) and at two pH values (pH 2 and pH 7), as summarized in Table 2. In each experiment, 0.1 g of BSY was added to the tartrazine solution, and the suspensions were maintained at the selected temperature until adsorption equilibrium was reached. After equilibrium, the samples were centrifuged, and the residual tartrazine concentration in the supernatant was determined by UV–vis spectrophotometry at 425 nm.

The thermodynamic evaluation was based on the apparent Gibbs free energy change (Δ*G*_app_), calculated from adsorption equilibrium data, to assess the favorability of the adsorption process at different temperatures and pH values. In heterogeneous adsorption systems involving biosorbents, such as inactivated yeast, the adsorption equilibrium constant derived from concentration ratios does not strictly correspond to a dimensionless thermodynamic equilibrium constant. Therefore, the calculated Gibbs free energy values should be interpreted as apparent quantities rather than absolute thermodynamic parameters.

The apparent Gibbs free energy change was calculated according to eq 4:

$$\Delta G_{\mathrm{app}}=-RT\ln K_{\mathrm{app}}$$ 4

where *R* is the universal gas constant, *T* is the absolute temperature, and *K*_app_ is an apparent equilibrium parameter derived from adsorption data. This approach allows a qualitative evaluation of the energetic favorability of adsorption, while avoiding overinterpretation of enthalpic and entropic contributions, which are often ambiguous in nonideal and heterogeneous adsorption systems.

Based on the Δ*G*_app_ values obtained, the adsorption of tartrazine onto BSY was found to be thermodynamically favorable at all studied temperatures and pH conditions, with more negative values observed under acidic conditions (pH 2), indicating stronger adsorbate–adsorbent interactions. Further mechanistic insights were obtained from complementary analyses, including adsorption isotherms, surface charge characterization, and kinetic modeling, rather than from enthalpy–entropy decomposition.

#### 2.2.3 Characterization of Tartrazine Dye Adsorbed on BSY

##### 2.2.3.1 Impact of pH and Temperature on the Stability of Dye Adsorbed on BSY

Four parallel experiments were conducted, each consisting of three replicated solutions. In each replicate, 0.1 g of inactivated brewer’s yeast (BSY) was added to 50 mL of buffer containing tartrazine at 10 mg·L^–^^1^. These samples were incubated at 10, 25, 37, and 90 °C. Throughout the experiment, the samples were protected from light and maintained under strictly monitored conditions appropriate to each temperature.

The amount of dye adsorbed by BSY was quantified by measuring the absorbance of the supernatant at 425 nm using a UV–vis spectrophotometer (model V-M5-BEL). Dye concentrations were calculated from calibration curves constructed with known standards, allowing the determination of adsorption capacity under each experimental condition.

#### 2.2.4 Release of Dye Adsorbed on BSY under Simulated Gastrointestinal Conditions

The release of adsorbed tartrazine was evaluated by exposing solutions containing adsorbed tartrazine on BSY to conditions simulating the basic conditions of the gastrointestinal tract. Twelve tubes, each containing 50 mL of tartrazine dye (10 mg·L^–1^) and 0.1 g of dry BSY, were prepared and incubated at pH 2 (25 °C) and pH 7 (37 °C) for 2 h. After centrifugation (5000*g*, 10 min), the precipitate was transferred to buffer solutions at pH 2 or pH 7 (HCl or KH_2_PO_4_) and incubated at 37 °C under agitation. Samples of 3 mL were collected at 5, 10, 20, 30, 40, 50, 60, 70, 80, 90, 100, and 120 min and analyzed using a UV–vis spectrophotometer (model V-M5-BEL) at 425 nm to determine the release of the dye from the BSY.

##### 2.2.4.1 Scanning Electron Microscopy (SEM)

To analyze the surface morphology and elemental composition of brewer’s yeast (BSY), a 100 mg sample was dispersed onto a metal substrate coated with conductive tape and palladium metallized to ensure conductivity. The analyses were performed using a FEI Quanta 400 Scanning Electron Microscope equipped with energy-dispersive spectroscopy (EDS) at the Mineral Technology Center (CETEM) of the Federal University of Rio de Janeiro. The sample was examined at magnifications ranging from 800× to 1600×.

For BSY analysis after tartrazine adsorption, samples were prepared by dispersing 0.1 g of inactivated yeast in 50 mL of tartrazine solution at 10 mg·L^–1^, under two pH conditions (pH 2 and pH 7). The mixtures were stirred for 2 h, then centrifuged at 5000*g* for 10 min. The supernatant was discarded, and the resulting biomass was collected, filtered on filter paper, and dried in an oven at 100 °C. The dry biomass samples were then stored appropriately before being subjected to SEM and EDS analysis using the same equipment and conditions described above.

##### 2.2.4.2 Fourier Transform Infrared Spectroscopy and Thermogravimetric Analysis

The characterization of the isolated synthetic dye (tartrazine), the isolated BSY biosorbent, and the dye adsorbed onto the BSY surface was performed using Fourier transform infrared (FTIR) spectroscopy and thermogravimetric analysis (TGA).

For the isolated tartrazine dye, FTIR spectra were obtained using a Spectrum 100 PerkinElmer spectrometer with a resolution of 4 cm^–1^ and 45 scans, within the spectral range of 4000–500 cm^–1^. The analyses were carried out using 1 g of pure tartrazine powder. TGA was performed using a TGA-50 instrument (Shimadzu, Kyoto, Japan). Approximately 10 mg of the sample was weighed into alumina pans, and the analysis was conducted under a nitrogen atmosphere (100 mL·min^–1^) at a heating rate of 10 °C/min, up to 1000 °C.

In the analysis of the isolated BSY biosorbent, FTIR spectroscopy was performed following the same procedure described for tartrazine, with the difference that dry brewer’s yeast biomass, previously dried in an oven at 100 °C, was used as the sample.

For the tartrazine-dyed BSY, both FTIR and TGA analyses were conducted using the same methodologies described above. The adsorption process was carried out using solutions at pHs 2 and 7, prepared with 10 mg·L^–1^ of the dye and 0.1 g of dehydrated brewer’s yeast biomass. After 2 h of contact time, the biomass with the adsorbed dye was collected, dried in an oven at 100 °C, and subsequently analyzed by FTIR and TGA.

## 3 Results and Discussion

### 3.1 Optimization of Adsorption Parameters

Adsorption of tartrazine onto inactivated *S. cerevisiae* (BSY) was performed using 0.1 g of biomass in 50 mL of solution for all experiments, ensuring a sufficient surface area for adsorption and reproducibility across conditions. Although systematic variation of the adsorbent dosage was not performed, this amount was chosen based on preliminary trials and literature values, as Banerjee et al.^19^ reported similar experimental conditions when optimizing the removal of tartrazine using sawdust as a low-cost biosorbent, evaluating parameters such as pH, contact time, and initial dye concentration.

Contact time was evaluated at pH levels of 2 and 7 for 5–120 min, with equilibrium achieved around 40 min. The tartrazine concentration was 10 mg/L, chosen based on the highest absorbance value still detectable by the spectrophotometer, ensuring an accurate reading without exceeding the instrument’s saturation limit. Similar concentrations were also employed by Madeira et al.,^20^ who investigated optimized adsorption conditions for tartrazine using activated carbon derived from biosolids, identifying a range of 10–20 mg/L as providing the most accurate and reproducible results.

The effect of pH was studied at 7 levels to simulate gastrointestinal conditions, and temperature was evaluated at 10, 25, 37, and 90 °C to assess thermal stability. These experiments enabled the identification of the primary parameters influencing adsorption and supported the selection of optimal conditions for maximum dye removal.

### 3.2 Physicochemical Characterization of Tartrazine Yellow Dye

#### 3.2.1 Solubility

Seven calibration curves were obtained and used as the basis for adsorption capacity tests and for analyzing the PCZ at various pH values (see Supporting Information for details). Due to their stability and representativeness, the curves at pH 2 and pH 7 were used as references for all subsequent analyses. These two pH values were selected to represent the acidic pH of the stomach and the basic pH of the intestine and help evaluate the structural and interaction changes of the dye and yeast more effectively.

The absorbance profiles at pH 2 and pH 7 showed linear behavior with increasing tartrazine concentration, indicating adherence to the Beer–Lambert law. Notably, higher absorbance values were observed at pH 7, as reflected by the steeper slope (0.1322) compared to pH 2 (0.1111), suggesting enhanced dye stability or ionization under neutral conditions. Additionally, the *R* ^2^ value at pH 7 (0.9972) was higher than that at pH 2 (0.976), reinforcing the influence of pH on the dye’s optical properties.

This difference may be attributed to tartrazine being more electronically stable at neutral pH. In contrast, at pH 2, the highly acidic environment can lead to protonation of functional groups (such as sulfonate or azo groups), altering the electronic distribution and π–π conjugation within the molecule. These changes can affect the chromophore structure and reduce the absorption efficiency, despite the higher degree of ionization under acidic conditions, as previously described by Kuschel et al.,^21^ who demonstrated that tartrazine undergoes distinct acid–base equilibria depending on the medium’s pH, influencing its electronic configuration and spectral properties.

The final concentration used in each curve was chosen based on the highest absorbance value still detectable by the spectrophotometer, ensuring accurate reading without exceeding the linearity limit of the equipment, as seen in the work by Silva et al.,^22^ where the curve goes up to 100 mg·L^–1^, the absorbance was high. This choice was crucial to ensure the reliability of the data obtained and to facilitate comparison between the different adsorption profiles of tartrazine on inactivated BSY.

### 3.3 Physical and Chemical Characteristics of the Dye and BSY, Based on the Analysis of FTIR Spectroscopy and TGA

FTIR and TGA were employed to characterize the pure tartrazine dye, the inactivated BSY, and the dye–biosorbent system after the adsorption process. These analyses aimed to investigate the functional groups present in each material and to identify possible physicochemical interactions between tartrazine and BSY.

#### 3.3.1 Analysis of Tartrazine Functional Groups

To assess the initial purity of the dye and detect possible degradation during adsorption onto BSY, the FTIR spectrum of tartrazine showed the preservation of its characteristic bands (Figure 1).

**Figure 1:**
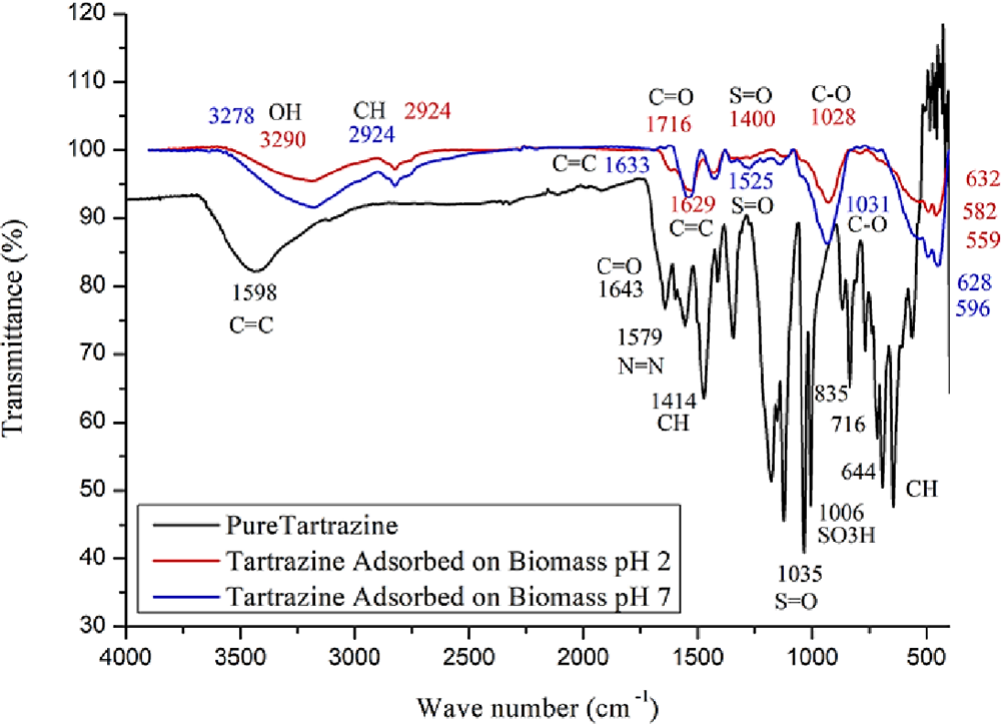
FTIR analysis of pure tartrazine dye and tartrazine dye adsorbed on yeast at pH 2 and pH 7.

The band at 1598 cm^–1^ is attributed to the stretching vibrations of C=C bonds in aromatic rings, common in compounds with benzene structures, mono- and disubstituted. The peaks at 1643 and 1579 cm^–1^ correspond to the asymmetric and symmetric vibrations of the azo bond (−N=N−), confirming the conjugation of the aromatic-azo system responsible for the intense coloration of the compound. Additionally, the band at 1414 cm^–1^ is related to the angular deformations in the plane (δ-CH) of aromatic hydrogens, which reinforces the presence of benzene nuclei in the molecule.^23^

The absorptions at 1006 and 1035 cm^–1^ are attributed to the symmetric and asymmetric stretching modes of the S=O bonds of the sulfonate group (−SO_3_^–^), indicating the chemical functionalization that confers solubility in water.^24^ Bands at 835, 716, and 644 cm^–1^ correspond to out-of-plane deformations (γ-CH) of aromatic hydrogens, typical of substituted aromatic systems, corroborating the chemical structure of tartrazine.^25^ Studies such as that by Tejada-Tovar et al.,^26^ which used FTIR, also reported minor changes in these characteristic bands during adsorption, confirming interactions between the functional groups of tartrazine and the biosorbent surfaces.

In particular, the presence of sulfonate groups and their characteristic bands is directly associated with the solubility of the dye in aqueous environments. At the same time, the aromatic–azo conjugated system contributes to its planarity and electronic delocalization, thereby affecting the color intensity and solvent interactions.^24,25^ Therefore, the vibrational data highlight the presence and behavior of specific functional groups characteristic of the dye’s molecular structure, confirming its chemical identity and integrity.

#### 3.3.2 BSY Functional Groups

This analysis was used to identify the functional groups present in pure BSY powder, revealing a complex composition. The band at 3271 cm^–1^ corresponds to hydroxyl (OH) groups, which are typical of carbohydrates, proteins, and polysaccharides. Bands at 1398 and 2916 cm^–1^ indicate C–H stretching vibrations associated with lipids and polysaccharides. The 1633 cm^–1^ band corresponds to carbonyl (C=O) groups linked to amide groups in proteins in the cell wall. Aromatic C=C vibrations at 1583 cm^–1^ suggest the presence of aromatic compounds. The 1228 cm^–1^ band is attributed to amines in proteins, and 1018 cm^–1^ is linked to C–O in polysaccharides like glucans. Finally, bands between 669 and 536 cm^–1^ correspond to aromatic amino acid residues.^17^

The FTIR analysis of BSY solubilized at different pH values (2.0 and 7.0) revealed several characteristic bands corresponding to distinct functional groups. The FTIR spectra obtained under two pH conditions, acidic (pH 2) and neutral (pH 7) (Figure 2A,B), show that the variation in pH did not lead to significant changes in the functional groups or chemical bonds within the BSY.

**Figure 2:**
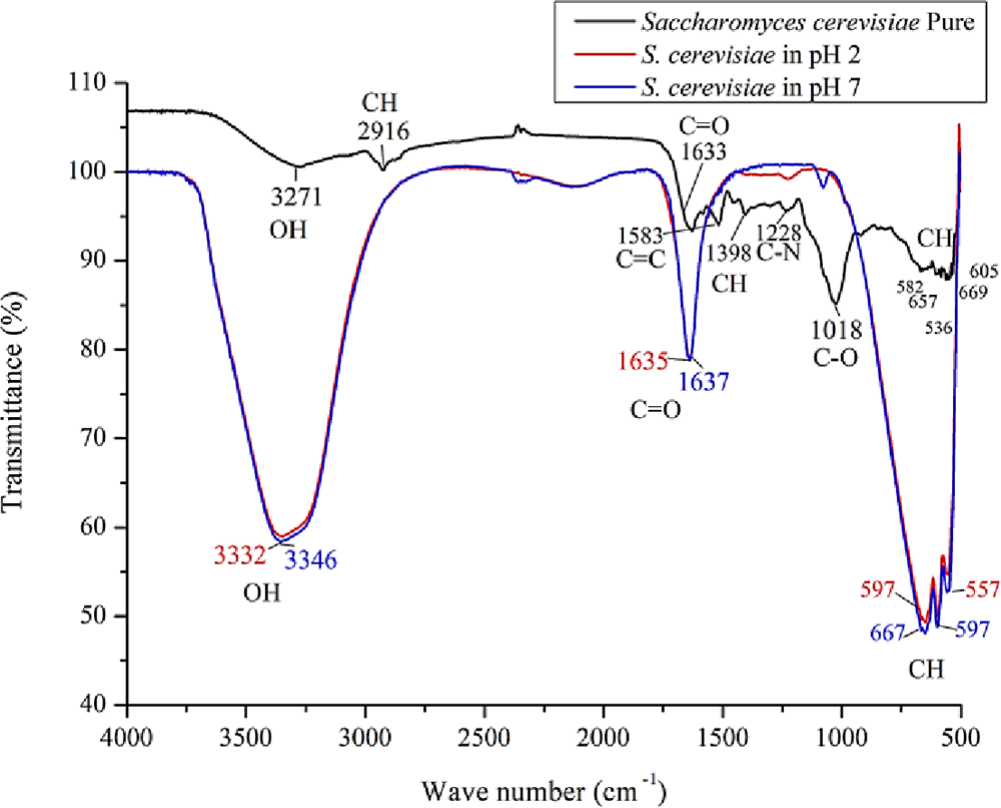
FTIR spectra of BSY and pure powdered and solubilized yeast at different pH values (2.0 and 7.0).

In the range of 3346 (pH 2) and 3332 cm^–1^ (pH 7), bands were observed that are attributed to the stretching vibrations of hydroxyl (OH) groups, associated with cell wall compounds such as carbohydrates, as well as other cellular metabolites.^26,27^ A band around 1635–1637 cm^–1^ was present under both pH conditions and related to carbonyl (C=O) groups. This absorption can be attributed to amides in proteins (such as those in the cell wall) and carbonyl groups in lipids that remain within the cells even after inactivation.^17^ Finally, the bands between 667 and 549 cm^–1^ are associated with out-of-plane C–H deformation vibrations in aromatic rings. These bands are typical of aromatic amino acid residues such as phenylalanine, tyrosine, and tryptophan, which are commonly found in cell wall proteins and other aromatic cellular components. Although slight variations in band positions were observed between the two pH conditions (667, 557, and 597 cm^–1^ at pH 2; 597, 557, and 549 cm^–1^ at pH 7), these differences are minimal and do not indicate significant structural alterations.^28^

Similar observations were reported in previous studies. Sartori^17^ identified a comparable OH-related band in pure yeast at 3271 cm^–1^, attributed to hydroxyl groups associated with carbohydrates in the cell wall. Additionally, vibrations at 1398 and 2916 cm^–^^1^ were assigned to C–H stretching, likely arising from aliphatic chains in lipids (such as methyl and methylene groups) and polysaccharides, particularly due to chemical modifications in mannans and glucans that can incorporate methyl groups. Ami^29^ also reported bands at 1583 and 1633 cm^–1^, associated with carbonyl (C=O) stretching, which were interpreted as signals of amide groups, indicating the presence of intact protein structures within the yeast cell wall. Furthermore, bands observed between 536 and 669 cm^–1^ previously identified in the analysis of pure tartrazine as corresponding to C–H out-of-plane deformations in aromatic rings were, in this context, attributed to aromatic amino acid residues such as phenylalanine and tyrosine.

The data indicate that BSY retains a complex chemical composition, comprising proteins, lipids, carbohydrates, and aromatic compounds. The persistence of characteristic bands across various pH conditions highlights the material’s structural robustness, underscoring its potential for applications in processes that involve pH variations without compromising its chemical integrity.

#### 3.3.3 Apparent Functional Groups in BSY Dye Adsorption

Figure 1 presents the FTIR analysis of tartrazine dye adsorbed on BSY at pH 2 (A) and pH 7 (B). The study at both pH 2 and pH 7 revealed characteristic bands related to the interaction between tartrazine dye and the yeast surface. The band at 3290 (pH 2) and 3278 cm^–1^ (pH 7) corresponds to the stretching vibration of −OH groups present in the yeast, indicating hydrogen bonding formation between the yeast hydroxyl groups and the functional groups of tartrazine.^17^

The band at 2924 cm^–1^, associated with C–H stretching of −CH_3_ and −CH_2_ groups in the yeast, remained unchanged between the two pH values, suggesting stable interactions with lipid components of the yeast cell membrane. In contrast, the bands at 1629 cm^–1^ (pH 2) and 1633 cm^–1^ (pH 7), attributed to C=C stretching in the aromatic structure of tartrazine, showed a slight shift, indicating pH-dependent variations in π–π interactions between the dye and the yeast. The band at 1716 cm^–1^ (pH 2), attributed to the C=O stretching of carbonyl groups, indicates interaction with the sulfonate or azo (−N=N−) groups of tartrazine but was not observed at pH 7, suggesting a reduced interaction at this pH.^17^

The bands at 1527, 1400, and 1226 cm^–1^ (pH 2) and 1525, 1371, and 1240 cm^–1^ (pH 7), corresponding to out-of-plane deformations of aromatic bonds (−SO_3_), indicate affinity between the aromatic structures of tartrazine and yeast, with slight changes in the interactions depending on the pH. The bands at 1028 (pH 2) and 1031 cm^–1^ (pH 7), associated with CO stretching in yeast carbohydrates or proteins, reflect the interaction between the sulfonate groups of tartrazine and biomass components, with a slight shift indicating changes due to adsorption.

Aragaw and Bogale^30^ identified similar functional groups in their FTIR study on biomass-based adsorbents used for dye removal from aqueous media. Their research demonstrated that hydroxyl (−OH), carbonyl (C=O), and sulfonate (−SO__3__^–^) groups play a critical role in adsorption processes through hydrogen bonding and electrostatic interactions between dye molecules and the biosorbent surface. The authors also observed that changes in the absorption bands, particularly in the 3300–1600 cm^–1^ region, are indicative of the active participation of surface hydroxyl and carboxyl groups in binding dye molecules. These findings support the interpretation presented in this work, which reported similar spectral changes during the adsorption of tartrazine onto *S. cerevisiae* biomass. Thus, these results support the hypothesis that the biosorption of anionic dyes, such as tartrazine, by yeast surfaces involves complex interactions among hydrogen bonds, π–π stacking, and electrostatic attraction, mechanisms that are sensitive to environmental conditions, including pH and temperature.

Finally, the bands at 632, 582, and 599 cm^–1^ (pH 2) and 628, 596, and 551 cm^–1^ (pH 7), attributed to deformation movements in the aromatic substituent groups, reinforce the interactions between the aromatic rings of tartrazine and yeast.^31^ These band variations suggest that pH influences interactions during adsorption, thereby affecting their dynamics and intensity.

#### 3.3.4 TGA of Tartrazine Dye

TGA (0–1000 °C) of pure tartrazine was performed to evaluate the dye’s mass loss and thermal stability. As shown in Figure 3, the thermal analysis of tartrazine dye demonstrated a gradual mass loss with several distinct stages. The first significant mass loss occurs between 0 and 100 °C, corresponding to the absorption of water, characteristic of the dye’s hygroscopic nature.^32^

**Figure 3:**
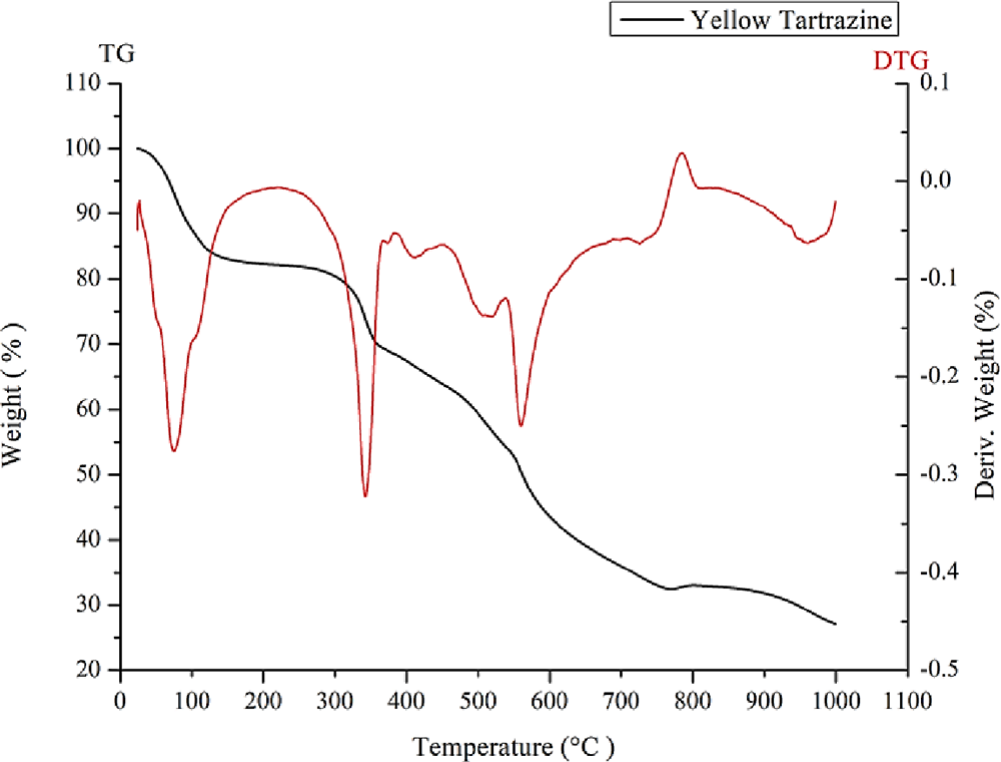
TGA (0 −1000 °C) of pure tartrazine.

Between 300 and 400 °C, a more notable mass loss is observed, indicating the pyrolytic thermal decomposition of the tartrazine molecule under inert conditions. This stage is characterized by the breakdown of key functional groups, such as the azo group (−N=N−), and is accompanied by an exothermic reaction. During this process, the azocyclic compound undergoes intermediate formation of azoxy species (−N­(O)=N−) before complete fragmentation.^33^

In the range of 450–500 °C, the gradual decomposition of additional molecular groups in tartrazine is observed, including the sulfonate groups (−SO_3_^–^), which are reported to be lost in this temperature range in the work of Leulescu.^24^ This is followed by an accelerated thermal degradation between 500 and 600 °C, possibly associated with the combustion of carbonaceous fragments generated during the earlier stages of the process.

Above 600 °C, the exothermic oxidation of residual carbon indicates the formation of inorganic ash and the complete breakdown of the remaining polycyclic aromatic nuclei. Finally, between 800 and 1000 °C, thermodynamic stability is reached, corresponding to the residual decomposition of these carbonaceous materials under extreme conditions. These stages underscore the sensitivity of tartrazine dye to temperature and its propensity for sequential degradation, beginning with more reactive functional groups (−N=N– and −SO_3_^–^) and progressing to complete decomposition.

The limit range at which the dye began to lose mass was 300 °C, after the loss of water at the initial temperatures. In addition, other factors contributed to the degradation, such as the controlled environment (nitrogen atmosphere), indicating that only mass loss due to heat occurred.

#### 3.3.5 TGA of Tartrazine Adsorbed onto BSY

The TGA was performed to observe the mass loss behavior associated with the adsorption of tartrazine dye onto BSY, providing information on the stability of the dye-biosorbent complex (Figure 4).

**Figure 4:**
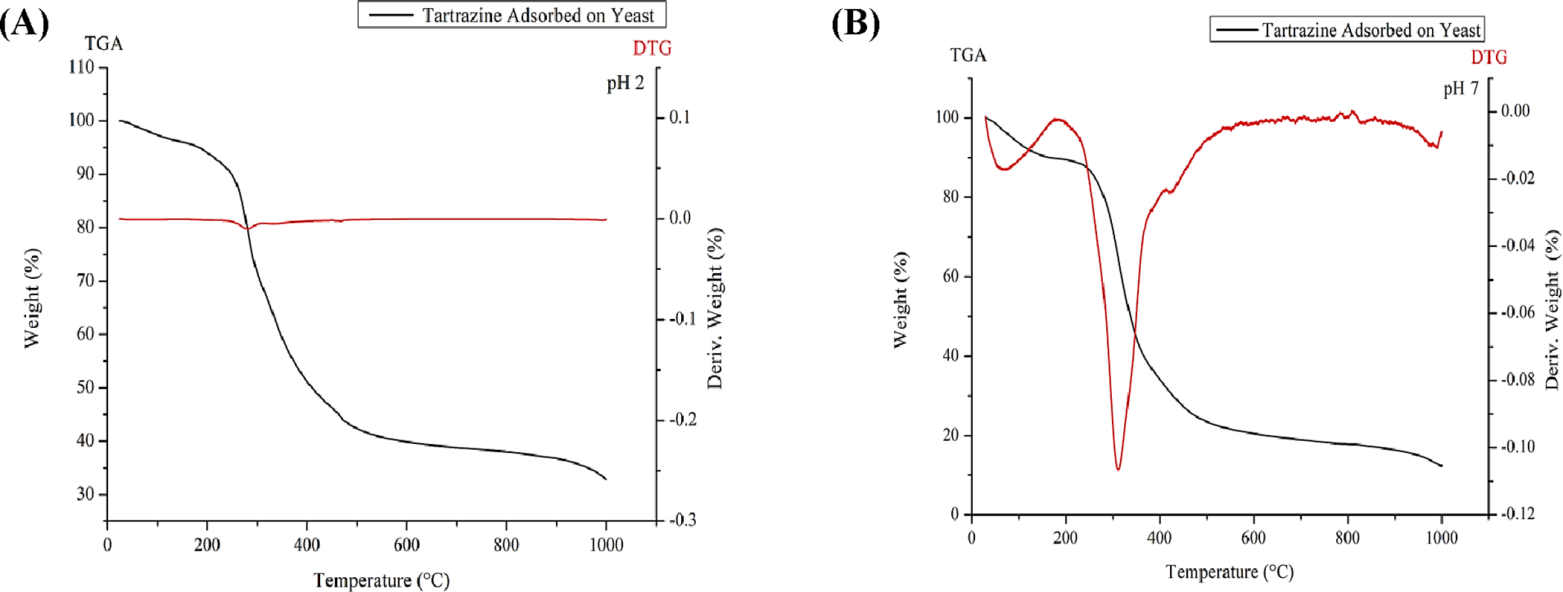
Thermoanalytical curve of tartrazine dye adsorbed on BSY at pH 2 (A) and pH 7 (B).

This is particularly important when alternative adsorbents, such as BSY, are evaluated as novel materials for dye adsorption, with potential applications in the food, beverage, cosmetics, and other industries. For BSY under acidic conditions (pH 2), moisture is released between 0 and 100 °C, followed by a stable phase up to 200 °C. From 200 °C onward, mass loss occurs, likely due to the decomposition of yeast components and their interactions with tartrazine.^34^

Between 450 and 600 °C, mass loss continues, possibly due to the degradation of yeast cell walls and tartrazine. At a neutral pH (7), moisture is initially released, and degradation begins between 300 and 400 °C, followed by the breakdown of organic components. From 450 to 500 °C, the mass loss rate slows, continuing up to 800–900 °C, where it likely forms carbonized residues.

In comparison with the analysis of the dye alone, the interaction of tartrazine with the biological matrix alters its thermal behavior, favoring temporary preservation of structural components and delaying specific thermolytic processes of the dye. The presence of refractory mineral residues at the end suggests that the yeast cell matrix plays a role in the final stabilization of the adsorbed system. Additionally, the more gradual mass loss under this condition, along with the reduced presence of DTG peaks, indicates a less abrupt decomposition process.

### 3.4 Physicochemical Behavior of the Pure Dye and in the Adsorption Process at Varying pH and Temperature

#### 3.4.1 Influence of Temperature on Dye Stability

The analysis of temperature effects on tartrazine was conducted by preparing solutions of the dye at different pH values and temperatures to evaluate its stability and behavior in solution. This assessment helps predict its potential applications under various storage and processing conditions. Figure 5 presents the variation in tartrazine absorbance over time at pH 2 (A) and pH 7.0 (B) under different temperature conditions, illustrating how both pH and temperature influence the dye’s stability in solution. Figure 5 indicates that at pH 2.0, the solubility of tartrazine is more stable at lower temperatures (10 and 25 °C), maintaining a constant concentration in solution, suggesting that the dye remains fully dissolved and that the absorbance follows the linear relationship predicted by the Beer–Lambert law.^35^

**Figure 5:**
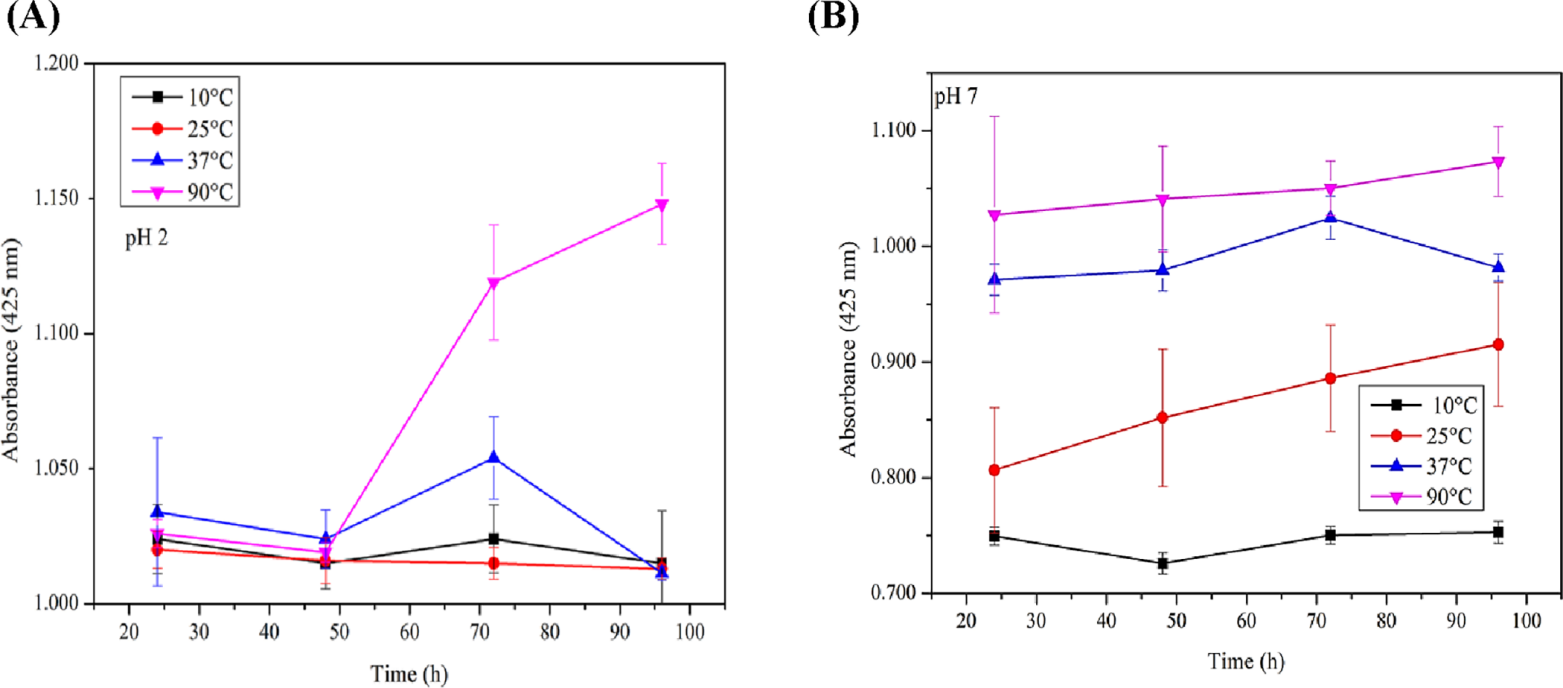
Variation of absorbance at 425 nm of tartrazine dye at pH 2 (A) and pH 7.0 (B) over time under different temperatures. Experimental conditions: 0.1 g of inactivated BSY was added to 50 mL of buffer solution containing tartrazine dye at a concentration of 10 mg·L^–1^. Samples were incubated at 10, 25, 37, and 90 °C.

However, at 37 °C, an oscillation in absorbance over time is observed, indicating that the dye’s solubility is affected by the increase in temperature at acidic pH. This variation in solubility interferes with absorbance since the Beer–Lambert law assumes complete dissolution of the solute. Thus, any change in solubility can lead to an apparent fluctuation in the dye absorbance measured by spectrophotometry, even if the actual amount of dye remains unchanged.^36^

At 90 °C, the absorbance increases sharply after 48 h, even in a closed system, suggesting that high temperatures, even in a stable acidic medium, induce a significant structural change in the tartrazine dye. This may indicate thermal degradation and the formation of byproducts that interfere with absorbance, and within its linearity range.

Although TGA indicates degradation only around 400 °C, continuous heating can cause thermal instability of the dye in solution. Compounds derived from this degradation, such as those resulting from the cleavage of the azo dye, including anilines (aromatic compounds with benzene rings), can form intermediate complexes that modify the observed absorbance.^35^ The absorbances determined at pH 7.0 (Figure 6B) were lower compared to pH 2.0 in the first 24 h.

**Figure 6:**
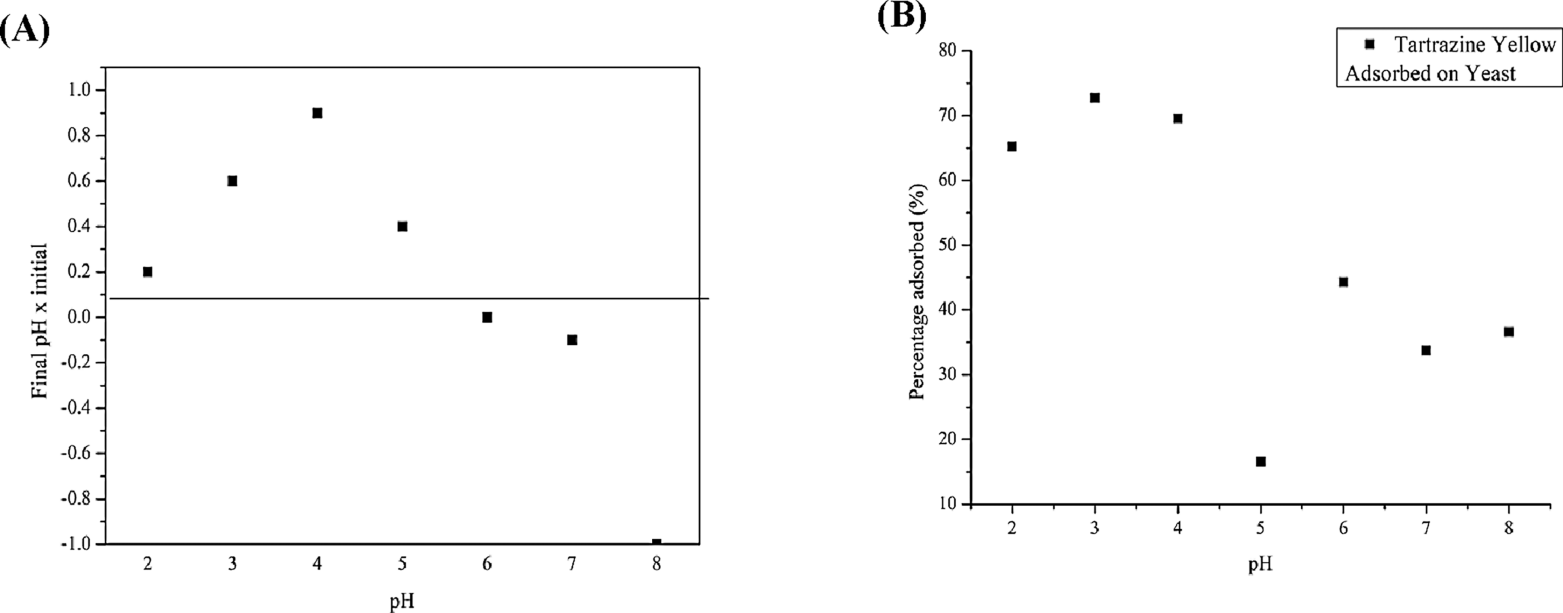
Tartrazine adsorbed on biomass with pH variations—PZC analysis (A)—and the relationship between pH and the percentage of tartrazine removal, highlighting the pH-dependent nature of the adsorption (B). Experimental conditions: Solutions containing 10 mg·L^–1^ of dye and 0.1 g of BSY were prepared at pH values ranging between 2 and 8 using McIlvaine buffer at room temperature (25 °C).

The possible reason for this reduction is deprotonation, which occurs in the neutral medium (pH 7) in the presence of an anionic dye. The loss of protons reduces the solution’s ionization and, consequently, its solubility, leading to slight precipitation on the container’s surface that interferes with the solution’s absorbance reading.

According to Lewis’s theory, which defines an acid as a species that accepts electrons and a base as a species that donates electrons, the behavior of tartrazine, an anionic dye, can be interpreted in the basic medium as undergoing deprotonation. This process leads to a less soluble conjugate base.^36^ Consequently, fewer molecules are available to absorb light, and the alteration of functional groups in the molecule also affects its light absorption properties in the UV–vis spectrum.

The behavior of the dye at pH 7.0 was more stable in terms of absorbance over time across all tested temperature ranges, indicating that tartrazine is more stable in this neutral medium, which promotes efficient dissolution. Nonetheless, lower absorbance values and reduced pigmentation were observed compared to pH 2, suggesting reduced chromophoric activity at neutral pH.

### 3.5 Adsorption of Tartrazine Yellow Dye on BSY

#### 3.5.1 Effect of pH on Dye Adsorption

Adsorbents can stabilize dyes, prevent their degradation, and extend the shelf life of formulations; therefore, it is essential to understand the adsorption capacity and stability of the adsorbent–dye complex. According to Čerovic,^37^ the surface charge of an adsorbent in aqueous solution depends on pH, as its active sites can gain or lose protons (H^+^ or OH^–^) through association or dissociation, depending on the adsorbent’s properties. One of the key properties for evaluating the adsorption potential of both BSY and the dye is the PZC, which is the pH at which the adsorbent surface carries no net electrical charge. Below this pH, the surface tends to be positively charged, while above it, it becomes negatively charged.

The PZC analysis indicated that at pH values between 2 and 5, the BSY surface was positively charged. This suggests that, under acidic conditions, the adsorbent tends to attract negatively charged species, such as the anionic dye tartrazine, thereby favoring its adsorption. At pH 6, the PZC was identified, indicating a neutral surface with no net charge. Above this point, at pHs 7 and 8, the BSY surface became negatively charged, which may favor the adsorption of cationic dyes but can lead to electrostatic repulsion of anionic dyes, such as tartrazine. To complement the PZC analysis, an additional test was conducted to assess the effect of pH on tartrazine adsorption. The results confirmed that adsorption behavior is strongly pH-dependent, supporting the role of electrostatic interactions between the dye and the adsorbent surface.

In addition to the PZC analysis, a complementary experiment was conducted to investigate the effect of pH on tartrazine adsorption onto BSY. By analyzing the percentage of dye adsorbed at different pH levels, it was possible to infer the nature of the interaction between the adsorbent and the dye, confirming the relevance of electrostatic forces in the process. Figure 6A presents the PZC analysis results, showing the surface charge behavior of the biomass across different pH values.

In contrast, Figure 6B illustrates the relationship between pH and the percentage of tartrazine removal, highlighting the pH-dependent nature of the adsorption.

At more acidic pH (2, 3, and 4), the adsorption efficiency of tartrazine ranges between 60 and 80%, primarily due to the electrostatic attraction between the opposite charges of the adsorbent and the adsorbate, as indicated by the PZC. As pH increases, efficiency decreases, reaching its lowest value at pH 5 (∼20%), where ionic competition and changes in the surface charge of the BSY negatively affect adsorption. This behavior is consistent with findings reported by Sartori^17^ for tartrazine encapsulated in alginate. At neutral (pH 7.0) and slightly alkaline (pH 8.0) conditions, adsorption remains low (30–40%) due to the negative charge of the BSY, which repels the anionic tartrazine. Additionally, competition with hydroxyl ions (OH^–^) interferes with the process, confirming that adsorption is more efficient under acidic conditions and is significantly reduced at neutral or alkaline pH.

Figure 7 is a visual representation of how the porous structure of the *S. cerevisiae* cell wall acts as a biosortive matrix for the tartrazine dye. The compartments shown suggest the three-dimensional organization of β-glucans, mannans, and structural proteins, which form a rigid yet permeable framework that allows the dye to diffuse to internal active sites.

**Figure 7:**
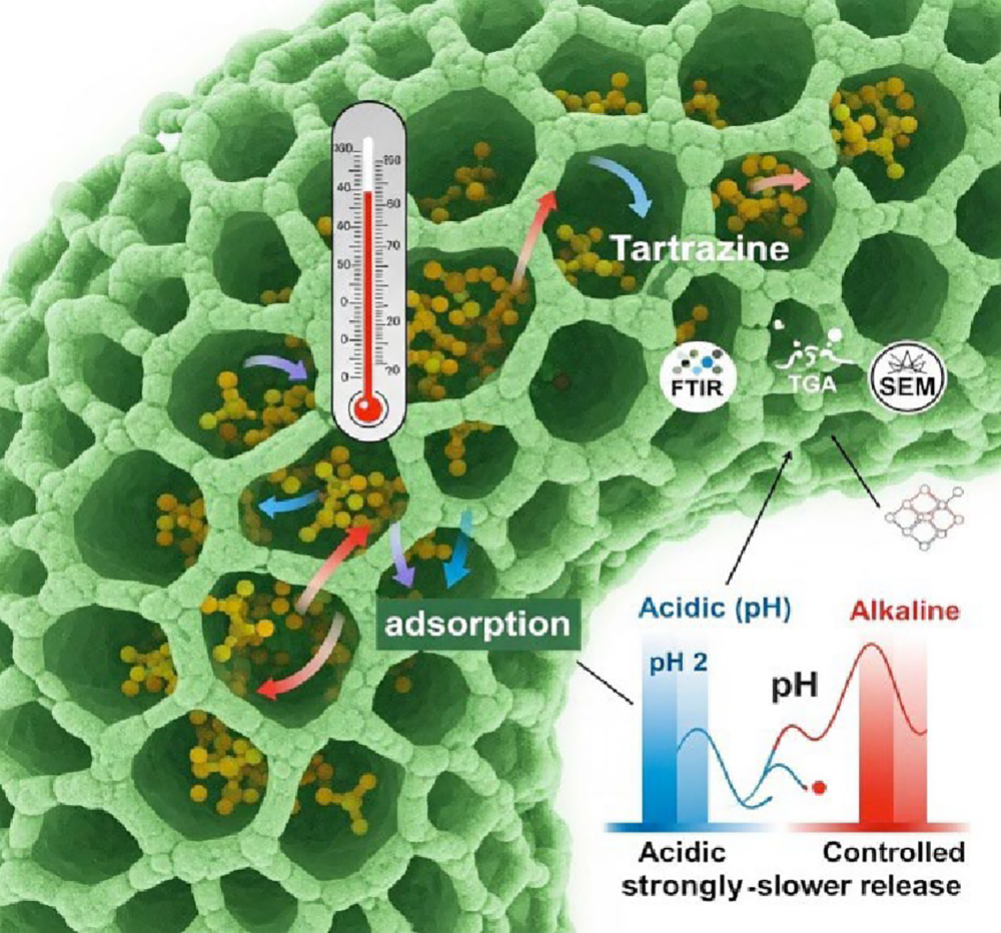
Electrostatic interaction between the yeast cell surface and tartrazine molecules. At a pH of 2, the yeast surface carries positive charges that attract the anionic tartrazine, thereby favoring adsorption. At a neutral pH of 7, the yeast surface charge is less favorable or neutral, leading to unfavorable interactions and reduced adsorption due to electrostatic repulsion with the anionic dye.

The process illustrated by arrows demonstrates pH-dependent adsorption, indicating that under acidic conditions, the dye has a greater affinity for the biomass. This is consistent with the ZCP results, which show that the yeast surface remains positively charged between pHs 2 and 5, thereby favoring electrostatic interactions with tartrazine, which contains negatively charged sulfonate groups. This result is similar to that obtained in Sartori’s work.^17^ The image reinforces this dynamic by showing greater dye retention in the region classified as “acidic pH,” which is in agreement with experimental removal values between 60 and 80% in this range, and the gradual release of the dye under basic conditions (“alkaline controlled release”), due to electrostatic repulsion between the negatively charged surface of the yeast and the dye, which corresponds to the behavior observed at pHs 7 and 8, where removal falls to 30–40%.

This behavior is also confirmed by SEM micrographs, which reveal apparent morphological differences between biomass exposed to acidic and neutral pH. At pH 2, the cell wall appears more irregular, suggesting a restructuring of the surface that increases the accessibility of adsorption sites. At pH 7, however, no changes in the surface were observed, consistent with the reduction in dye retention observed experimentally. These structural variations directly reinforce the pH-dependent adsorption mechanism illustrated in Figure 7.

In addition to electrostatic attraction, the figure highlights the heterogeneity of possible chemical interactions between biomass and dye. The symbols referring to FTIR analyses and the arrows indicating different bond directions suggest the presence of hydrogen bonds and π–π interactions between aromatic rings induced by temperature and pH. FTIR itself, as discussed in the text, confirms that bands related to −OH, C=O, amines, and C–O change adsorption, evidencing the involvement of these functional groups in the interaction. These spectroscopic changes are consistent with studies such as that by Aragaw and Bogale,^30^ which also identified the active participation of hydroxyls, carbonyls, and aromatic groups in the adsorption of anionic dyes by microbial biomass.

The thermometer in the image highlights the role of temperature in stability and adsorption behavior, as moderate temperatures tend to favor dye permeation into the cell wall. Still, under extreme conditions, such as near 90 °C, there are indications of structural alterations to the molecule, as suggested by the abrupt increase in absorbance observed experimentally in relation to pure tartrazine, which is reaffirmed in the test “Effect of temperature on the adsorption of tartrazine dye on BSY.”

Taken together, the image visually summarizes the biosorption phenomenon discussed throughout this paper: a multifactorial process that combines electrostatic interactions, functional affinities, thermal rearrangements, and pH influence, all modulated by the structural characteristics of the yeast cell wall.^19,20^

#### 3.5.2 Adsorption Kinetics and Isotherm Models

To evaluate the temporal behavior of the dye adsorption process on BSY, determining the time needed to reach equilibrium, as well as the adsorption rate and the mechanisms involved, adsorption kinetics were carried out at different pHs, using the most common kinetic models, such as pseudo-first order and pseudo-second order models (Figure 8).^38^ Equilibrium was reached after 40 min, with a pH of 2 and 7. However, the primary purpose of the kinetic test was to characterize the adsorption mechanism and adjust the model.

**Figure 8:**
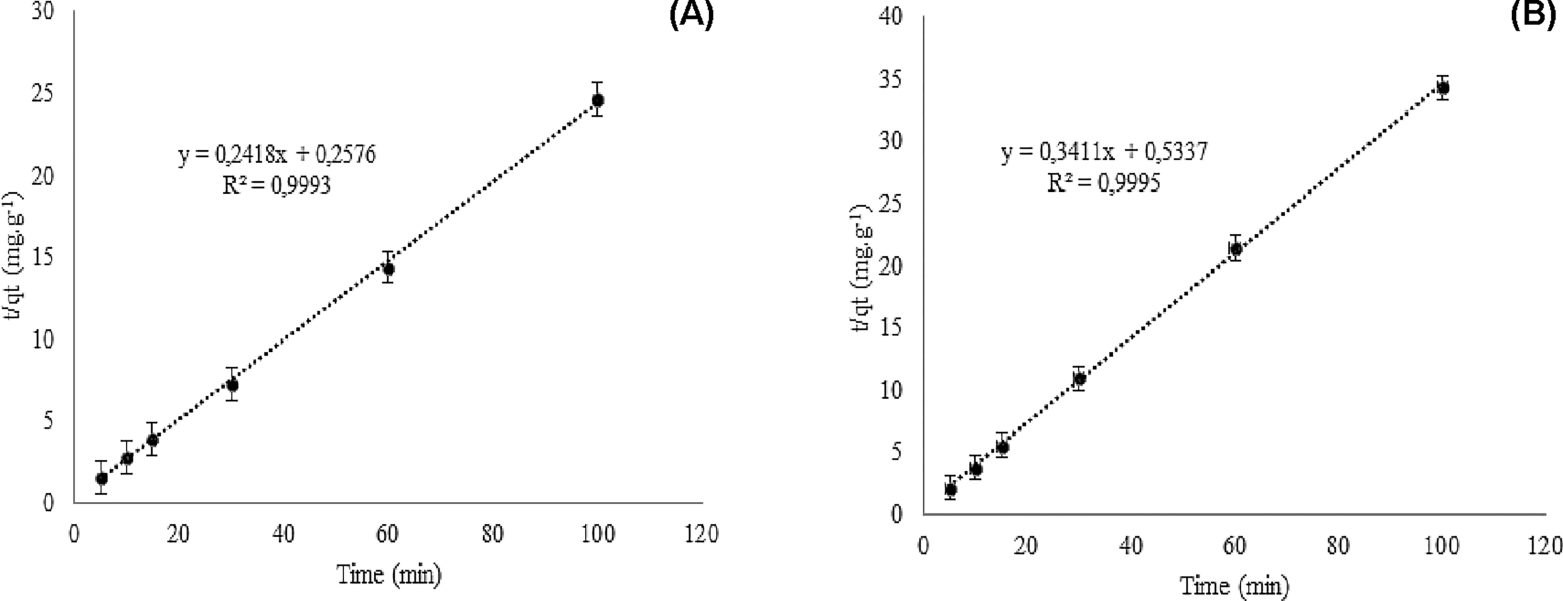
Kinetic linear regression model at pH 2 (A) and pH 7 (B), pseudo-second order. For the kinetic tests, 50 mL solutions of McIlvaine buffer at pH 2 and pH 7, to which 10 mg of tartrazine and 0.1 g of previously dried BSY (24 h at 105 °C) were added. The solutions were stirred continuously at room temperature (25 °C), and aliquots of 3 mL were collected at times between 5 and 120 min. After centrifugation (5000*g*, 10 min), the samples were analyzed by spectrophotometry UV–vis at 425 nm.

In the general comparison of the kinetic models of adsorption of the tartrazine dye in BSY, the pseudo-second-order model fit the experimental data, with high *R* ^2^ (*R* ^2^ > 0.99), indicating that the data fit well the model that suggests that chemical interactions control adsorption, chemisorption involves the formation of chemical bonds (covalent or coordinated), between adsorbate and adsorbent,^39,40^ being slower and generally irreversible, but resulting in more efficient and specific adsorption. Interpreting the effects of pH, comparing the adsorption kinetics at pHs 2 and 7, it was observed that at pH 2, the higher initial adsorption rate (*k*_2_) can be explained by the presence of protonated groups on the BSY, which interact more quickly with the dye, an anionic molecule with groups such as sulfonate, which are almost always ionized in a more acidic environment. This generates an attraction between the opposite charges present in the dye molecules and the surface pores of the BSY, resulting in better overall adsorption, as more dye is retained.^41^

The *k*_2_ at pH 7 indicates that the process is slower, possibly due to weaker electrostatic interactions between the adsorbent’s surface charge and the adsorbate, both of which are negative. However, these interactions are compensated by other forces such as hydrogen bonds and van der Waals forces.^42^ At pH 2, the high *Q*_*e*_ suggests that the acidic environment favors interaction between the tartrazine dye and the protonated functional groups of the BSY, with a maximum adsorbate adsorption of 4.14 mg·g^–1^ per gram of adsorbent. This means that, upon reaching equilibrium, each gram of the adsorbent material will be able to retain up to 4.14 mg of the adsorbate. At pH 7, the reduced *Q*_e_ of 2.93 mg·g^–1^ may be the result of a lower overall affinity, since the surface charge of the BSY and tartrazine dye may repel each other at neutral pH.

According to the work of Ho and McKay,^43^ the pseudo-second-order model, which relates the adsorption rate to the amount of dye already adsorbed, indicates saturation of the active sites, both on the surface and within the pores. Adsorption occurs on both the cell surface and within internal pores or other available adsorption sites. The external surface is typically the primary adsorption site due to physicochemical interactions, such as van der Waals forces, ionic interactions, and hydrogen bonds between functional groups.

An indirect comparison between the adsorption of Rose Bengal (1017 g/mol) and tartrazine (534 g/mol) on residual yeast was carried out to estimate the porosity of the yeast cell wall. Despite its larger molecular size, Rose Bengal exhibited higher adsorption (4.98 mg·g^–1^ at pH 2 and 5.00 mg·g^–1^ at pH 7), suggesting that interactions occur mainly within larger pores, favoring both chemical and physical adsorption. In contrast, tartrazine, being smaller, is likely to diffuse more rapidly. These results indicate that adsorption is primarily governed by chemical interactions rather than diffusion limitations, supporting the classification of the yeast structure as mesoporous (2–50 nm), as defined by the IUPAC.

#### 3.5.3 Isotherm

To investigate the effect of the initial dye concentration on adsorption capacity, equilibrium experiments were performed at different initial tartrazine concentrations (2.5–50 mg/L). These data were further analyzed using isotherm models.

According to Giles’ classification,^44^ the adsorption isotherm of the tartrazine dye at pH 2 (Figure 9A) is of the L2 type, characterized by a rapid initial growth of the adsorbed quantity (*Q*_e_) with increasing adsorbate concentration (*C*_e_), followed by a plateau. This indicates that the adsorption sites are rapidly occupied at the beginning, but as saturation approaches, the adsorption rate decreases.

**Figure 9:**
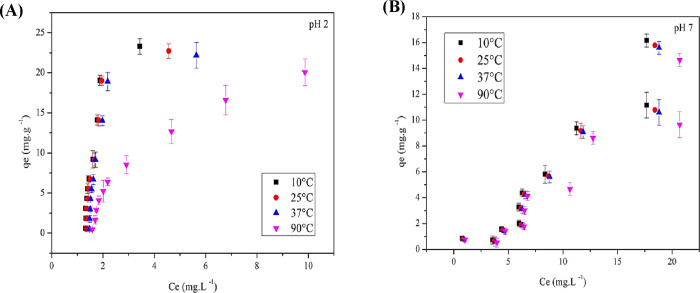
(A) Tartrazine yellow adsorption isotherm on residual yeast at pH 2 and (B) pH 7 (standard deviations magnified 100× for better visualization). For the isotherm tests, solutions containing 0.1 g of dry biomass and varying concentrations of tartrazine (2.5–50 mg·L^–1^) were prepared in triplicate (50 mL each), adjusted to pH 2 and pH 7, and maintained at temperatures of 10, 25, 37, and 90 °C. After centrifugation (5000*g*, 10 min), the samples were analyzed by UV–vis spectrophotometry at 425 nm to determine *Q*_e_ and *C*_e_ using eq 3, as described in the Materials and Methods.

Mendes et al.^45^ observed a similar behavior in the adsorption of Direct Orange 2GL dye by *S. cerevisiae* biomass, as well as in the sigmoidal form of the adsorption of tartrazine by sugarcane bagasse. The L2 curve suggests that adsorption is limited by the adsorbent’s maximum capacity and by the balance between adsorption forces and the adsorbate concentration. At lower temperatures (10 and 25 °C), a stable adsorption plateau is formed, whereas at higher temperatures, this plateau is not well-defined. At 37 °C, there may be available sites due to lower adsorption energy, while at 90 °C, dye retention is inefficient.

At pH 7 (Figure 9B), the isotherm is of the S4 type, indicating cooperative adsorption, where the initial adsorption facilitates the fixation of more molecules, increasing the surface affinity.

This sigmoidal behavior is common in heterogeneous surfaces and involves multiple adsorption layers and pore filling. Despite the rapid initial adsorption, final stability is reached without plateau formation, and performance is lower than at pH 2, where adsorption was more efficient. Baleeiro^46^ reported a sigmoidal isotherm for the adsorption of tartrazine on sugarcane bagasse. The adsorption kinetics confirm this trend, showing that the acidic medium favors overall adsorption, even though the initial step is slower.

Table 1 presents the parameters of the adsorption isotherms adjusted via nonlinear optimization to the equilibrium data of the tartrazine dye, adsorbed on BSY at 10, 25, 37, and 90 °C, as well as the values of the respective adjusted correlation coefficients.

**Table 1:** Parameters of Adsorption Isotherms of the Tartrazine Dye on BSY, pH 2

**model**	**parameters**	**10 °C**	**25 °C**	**37 °C**	**90 °C**
Temkin pH 2.0	*B* (kJ·mol)	0.09	0.13	0.17	0.29
	*k*_1_ (L·mg)	0.83	3.6	1.33	0.17
	*R* ^2^	0.9936	0.9938	0.9933	0.9926
Dubinin–Radushkevich pH 7.0	*q*_max_ (mg·g)	33.5	42.4	49.8	74.7
	*B* (mol·J)	1.79	2.03	2.20	2.60
	*E* (kj·mol)	0.53	0.50	0.48	0.44
	*R* ^2^	0.9709	0.9675	0.9646	0.9569

At pH 2, it can be concluded that the model that best suited each temperature was Temkin’s, 10 °C (*R* ^2^ = 0.9934), 25 °C (*R* ^2^ = 0.9933), 37 °C (*R* ^2^ = 0.9931), and 90 °C (*R* ^2^ = 0.9922), representing the equilibrium curves of the tartrazine dye on BSY. This model assumes that the adsorption energy decreases as the adsorbent surface becomes increasingly covered with adsorbate. The variation in the adsorption energy (*B*) increases with increasing temperature, indicating that higher temperatures favor adsorption. On the other hand, the adsorbent surface affinity (*k*_1_) increased from lower temperatures to ambient and elevated temperatures (25 and 37 °C). The *k*_1_ at 25 °C showed the best value, indicating the highest affinity at this temperature.

Temkin considers the interactions between adsorbent and adsorbate molecules, as well as the nonuniform adsorption process, in which different types of adsorption energy co-occur. This molecular interaction model is commonly observed in interactions involving chemisorption, as described in adsorption kinetics.^47^

At pH 7, the most suitable model was the Dubinin–Radushkevich model, yielding the highest *R* ^2^ values: 10 °C (*R* ^2^ = 0.9709), 25 °C (*R* ^2^ = 0.9675), 37 °C (*R* ^2^ = 0.9646), and 90 °C (*R* ^2^ = 0.9567). This model is characteristic of adsorption by micropores, where molecular interaction forces are weaker, such as physical interactions, without the formation of layers. It also characterizes adsorption in which the active sites are not saturated. The parameters *Q*_max_ (maximum adsorption capacity) and *B* (adsorption energy constant) increased with temperature, further supporting the idea that temperature enhances molecular interactions.^47^

#### 3.5.4 Thermodynamic Parameters of the Adsorption Process

The thermodynamic behavior of tartrazine adsorption onto BSY was evaluated at different temperatures (10, 25, 37, and 90 °C) and pH values (2 and 7), as summarized in Table 2. The analysis was based on the apparent Gibbs free energy change (Δ*G*_app_), calculated from adsorption equilibrium data, in order to assess the favorability of the adsorption process.

**Table 2:** Thermodynamic Parameters (Δ*G*°, Δ*H*°, and Δ*S*°) for the Adsorption of Tartrazine Dye on Inactivated BSY at Different Temperatures and pH

	temperature (°C)	Δ*G* (kJ mol^–1^)
pH 2	10	–13.85
	25	–13.76
	37	–13.70
	90	–13.41
pH 7	10	–4.30
	25	–4.15
	37	–4.03
	90	–3.49

Negative Δ*G*_app_ values were obtained at all temperatures for both pH conditions, indicating that tartrazine adsorption onto BSY is thermodynamically favorable. At pH 2, Δ*G*_app_ values ranged from −13.8 to −13.4 kJ mol^–1^, whereas at pH 7, the values were less negative, varying between −4.3 and −3.5 kJ mol^–1^. These results demonstrate a higher affinity of the adsorbent for the anionic dye under acidic conditions, consistent with the PZC analysis, which shows that at pH 2 the BSY surface is predominantly positively charged, favoring electrostatic attraction with tartrazine molecules.

The influence of temperature on Δ*G*_app_ indicates that adsorption is favored at moderate temperatures, with improved performance observed up to 37 °C. Deviations observed at 90 °C, particularly at pH 2, may be associated with partial structural instability of the biosorbent at elevated temperatures, which can affect adsorption efficiency.

Due to the heterogeneous nature of biosorbents and the nonideal character of adsorption equilibrium constants, the thermodynamic discussion in this study was restricted to apparent Gibbs free energy changes. In such systems, the direct interpretation of enthalpy and entropy contributions is often ambiguous and may not reliably reflect the underlying adsorption mechanism. Therefore, mechanistic insights were primarily derived from complementary analyses, including isotherm modeling, surface charge characterization, and kinetic studies.

The isotherm results support the thermodynamic trends observed, with the Temkin model providing the best fit at pH 2, suggesting strong adsorbate–adsorbent interactions with decreasing adsorption energy as surface coverage increased. At pH 7, the Dubinin–Radushkevich model showed better agreement with the experimental data, indicating a predominantly physical adsorption process involving weaker interactions within micropores.^18^ These findings are consistent with the kinetic analysis, which showed that adsorption followed a pseudo-second-order model, with higher adsorption rates at pH 2, suggesting stronger interactions between tartrazine molecules and the BSY surface.

Similar thermodynamic behavior has been reported by Amaku et al.^48^ for tartrazine adsorption using sawdust as an adsorbent, where negative Gibbs free energy values indicated favorable adsorption, reinforcing the validity of the trends observed in the present study.

Overall, the thermodynamic evaluation, combined with kinetic and isotherm analyses, indicates that tartrazine adsorption onto BSY is a favorable process, with stronger interactions occurring under acidic conditions. This behavior is primarily attributed to electrostatic attraction between protonated functional groups on the BSY surface and anionic tartrazine molecules, in agreement with surface charge and adsorption modeling analyses (Figure 10).^18^

**Figure 10:**
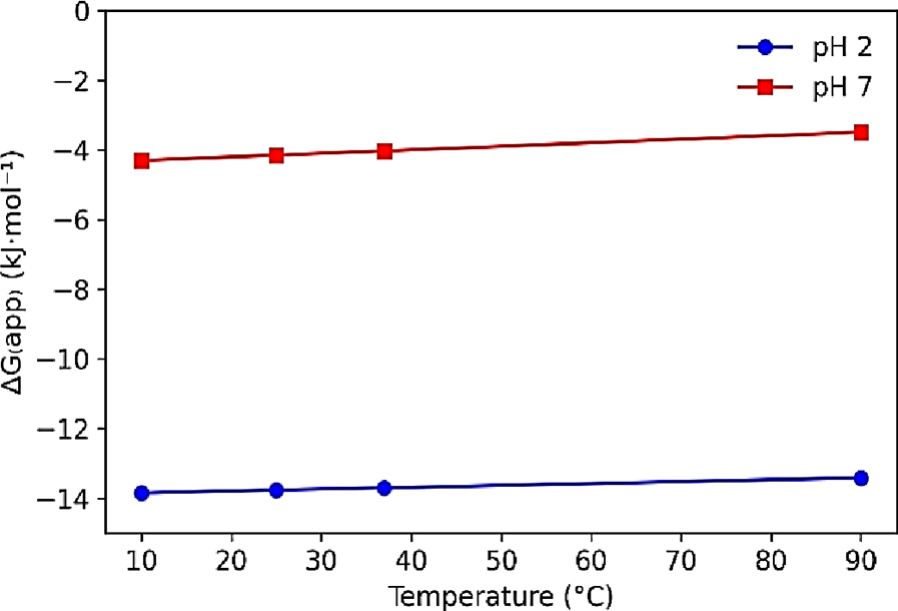
Effect of temperature on the apparent Gibbs free energy change (Δ*G*_app_) for tartrazine adsorption onto BSY at pH 2 and pH 7.

#### 3.5.5 Effect of Temperature on the Adsorption of Tartrazine Dye on BSY

Temperature significantly influences the adsorption process by increasing the kinetic energy and mobility of adsorbate molecules, as well as enhancing intraparticle diffusion.^49^ Therefore, determining the optimal temperature is essential for maximizing adsorption efficiency. Figure 11 presents adsorption as a function of different temperatures and pH levels of 2 (Figure 11A) and 7.0 (Figure 11B).

**Figure 11:**
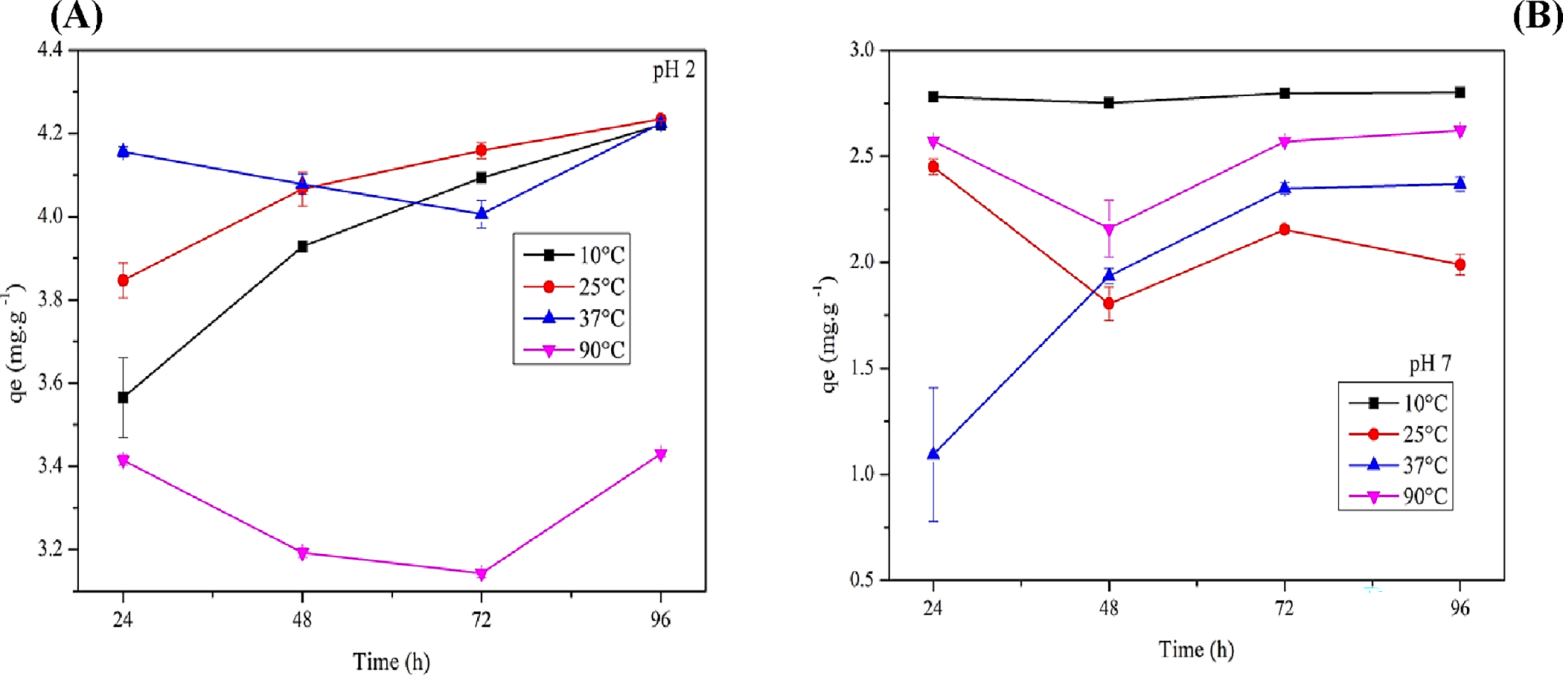
Adsorption as a function of time at different temperatures at pH 2 (A) and pH 7 (B). Experimental conditions: 0.1 g of inactivated BSY was added to 50 mL of buffer solution containing tartrazine dye at a concentration of 10 mg·L^–1^. Samples were incubated at 10, 25, 37, and 90 °C.

Upon observing the results of tartrazine dye adsorption at different temperatures and pH 2, distinct adsorption behaviors were observed. Among them, 25 °C showed faster adsorption than 10 °C. This can be attributed to the kinetic and isothermal characteristics, where the acidic pH and moderate temperature enhance molecular mobility and interaction between the adsorbent and adsorbate, resulting in greater overall dye adsorption on BSY.^50^

Despite differences in initial adsorption rates, samples at 10 and 25 °C stabilized after 72 h, indicating that BSY reaches a saturation point at which the amount of adsorbed dye remains constant, regardless of temperature. At 37 °C, however, a rapid initial adsorption was followed by fluctuations, suggesting desorption after 24 h. This behavior may result from the establishment of a dynamic equilibrium or competition between tartrazine molecules for active sites, leading to the release of dye back into the solution. Over prolonged periods, the interaction between the dye and the adsorbent appears to be less stable at higher temperatures.

According to Nascimento,^51^ adsorption equilibrium occurs when the molecules or ions of the adsorbate migrate from the aqueous medium to the adsorbent surface until equilibrium is established, at which point the solute concentration in the liquid phase (*C*_e_) remains constant. Observing the behavior at different temperatures, all reached adsorption equilibrium, as they achieved a final concentration close to 4.2 mg·g^–1^, with 25 °C showing the highest final adsorption value, although only slightly higher (4.23 mg·g^–1^).

Adsorption at 90 °C yielded less satisfactory results, characterized by a low initial capacity and irregular fluctuations over time. This behavior indicates that, at this constant temperature, the yeast may alter its essential chemical components for adsorption, thereby reducing the BSY’s affinity for tartrazine molecules. Consequently, the adsorption process becomes less efficient, leading to an unstable adsorption (3.4 mg·g^–1^), as observed in the adsorption isotherm and the tartrazine stability test.

At 10 °C and neutral pH, adsorption was efficient and reached equilibrium quickly, with minimal fluctuations. At 25 °C, adsorption started at a lower level and exhibited sharp variations, indicating a more sensitive equilibrium. At 37 °C, adsorption was gradual but consistent, with strong interactions maintaining balance. In contrast, at 90 °C, performance declined, with pronounced fluctuations in adsorption capacity. Adsorption was lower at neutral and basic pH levels, as indicated by the kinetics. The most significant initial adsorption occurred at 10 °C (2.7 mg·g^–1^). Still, at 37 °C, the process was more effective, with gradual, stable adsorption (2.37 mg·g^–1^), unlike at the lowest temperature, which showed no continuity in adsorption over the days.

pH 2 at 25 °C was the most favorable condition for tartrazine adsorption, as it combined rapid interaction and high final capacity. Although adsorption at pH 7 and 37 °C was more stable, it also exhibited higher initial and final adsorption levels, as this temperature provides sufficient thermal energy for molecular interactions and efficient diffusion. In contrast, pH 2 at 90 °C resulted in the lowest adsorption efficiency. At pH 2, the solution’s pH remained stable for the first 24 h, with only slight fluctuations thereafter.

After 48 h, pH increased at 25 °C (2.8) and 37 °C (2.9), suggesting more intense dye–adsorbent interactions. By 72 h, the pH had stabilized again, especially at 10 °C, whereas variations at 37 and 90 °C were less pronounced, indicating that temperature influences system dynamics, including ion adsorption and release. At pH 7, values remained stable at around 7 after 24 h. Over time, the pH increased slightly at 37 °C, while remaining relatively constant at 10, 25, and 90 °C. Compared to the PZC (pH ∼ 6), values between 6 and 7 suggest weak surface charge interaction and limited electrostatic adsorption, consistent with Khattri,^52^ who reported that temperature changes influence adsorption capacity.

#### 3.5.6 SEM of BSY

SEM was performed to evaluate the morphological characteristics of BSY before and after tartrazine dye adsorption. Figure 12 presents scanning electron micrographs that illustrate these structural features. Figure 12A,B represents the only BSY at magnifications of 800× and 1600×, respectively, where the cells appear agglomerated, maintaining their typical rounded shape.

**Figure 12:**
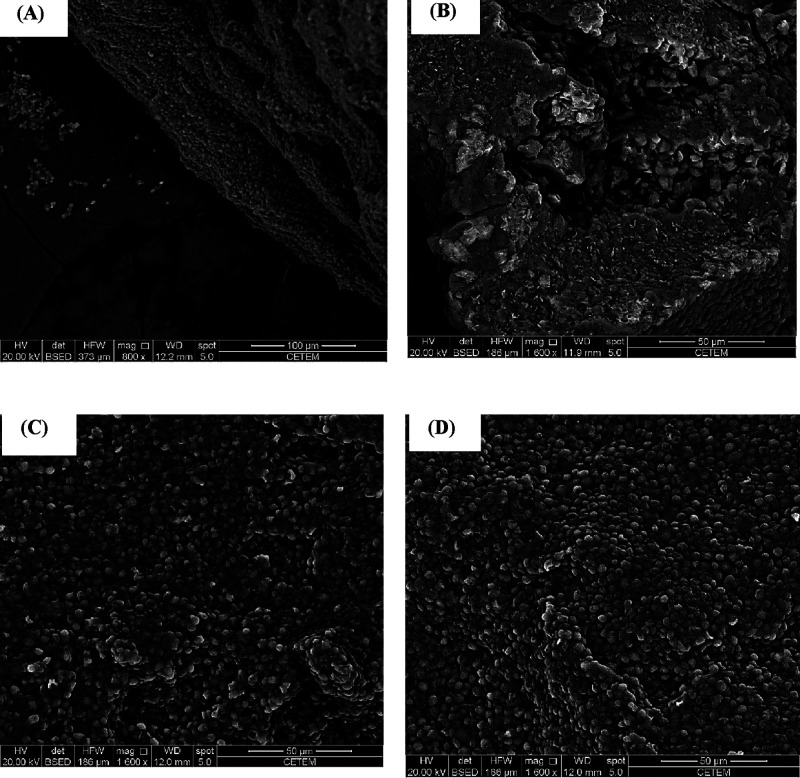
Scanning electron micrographs of only BSY and tartrazine dye adsorption: (A) BSY at 800× magnification, (B) BSY at 1600× magnification, (C) BSY after tartrazine adsorption at pH 2, and (D) BSY after tartrazine adsorption at pH 7.

Figure 12 C,D depicts BSY after tartrazine dye adsorption at pH 2 and pH 7, respectively. No significant changes in the overall physical structure of the biomass were observed; however, surface irregularities were more pronounced at pH 2. Conversely, at neutral pH 7, lower dye retention resulted in a more uniform surface appearance.

Due to the composition of the yeast cell wall and the presence of functional groups capable of ionization, the interaction between the dye and biomass is influenced by pH. In acidic conditions, the biomass surface becomes positively charged, as confirmed by the PZC test, favoring the retention of the anionic tartrazine dye. In contrast, under basic conditions, the negatively charged surface repels the anionic dye, resulting in weaker interactions and reduced adsorption. Similar patterns were reported by Mendes,^45^ supporting the influence of pH on dye distribution during adsorption.

### 3.6 Release of Dye Adsorbed on BSY under Simulated Gastrointestinal Conditions

The exposure of tartrazine-dyed BSY to simulated gastrointestinal conditions aimed to evaluate the material’s behavior in ingestion-like scenarios, enabling predictions about its stability in food products and whether, during digestion, the dye would remain adsorbed to the biomass or be released into the body. For this purpose, buffers simulating the gastrointestinal tract environment were used: pH 2 (HCl and NaCl) to represent the stomach, which is rich in chloride ions, and pH 7 (KH_2_PO_4_ and NaOH) to mimic the small intestine, where nutrient absorption occurs.^53^ The conditions selected for the simulation test were pH 2 at 25 °C and pH 7 at 37 °C, as these showed greater efficiency in tartrazine adsorption.

Figure 13 presents the simulated release profile of tartrazine dye adsorbed on BSY in gastric (A) and intestinal (B) environments. The results indicated that gastrointestinal conditions influenced the release of tartrazine from BSY. At pH 2 and 25 °C (stomach simulation with HCl + NaCl), the dye was gradually released, possibly due to competition between H^+^ ions and the dye for adsorption sites, leading to desorption. However, at pH 7 and 37 °C, the release was minor and more gradual, suggesting greater retention of tartrazine under these conditions.

**Figure 13:**
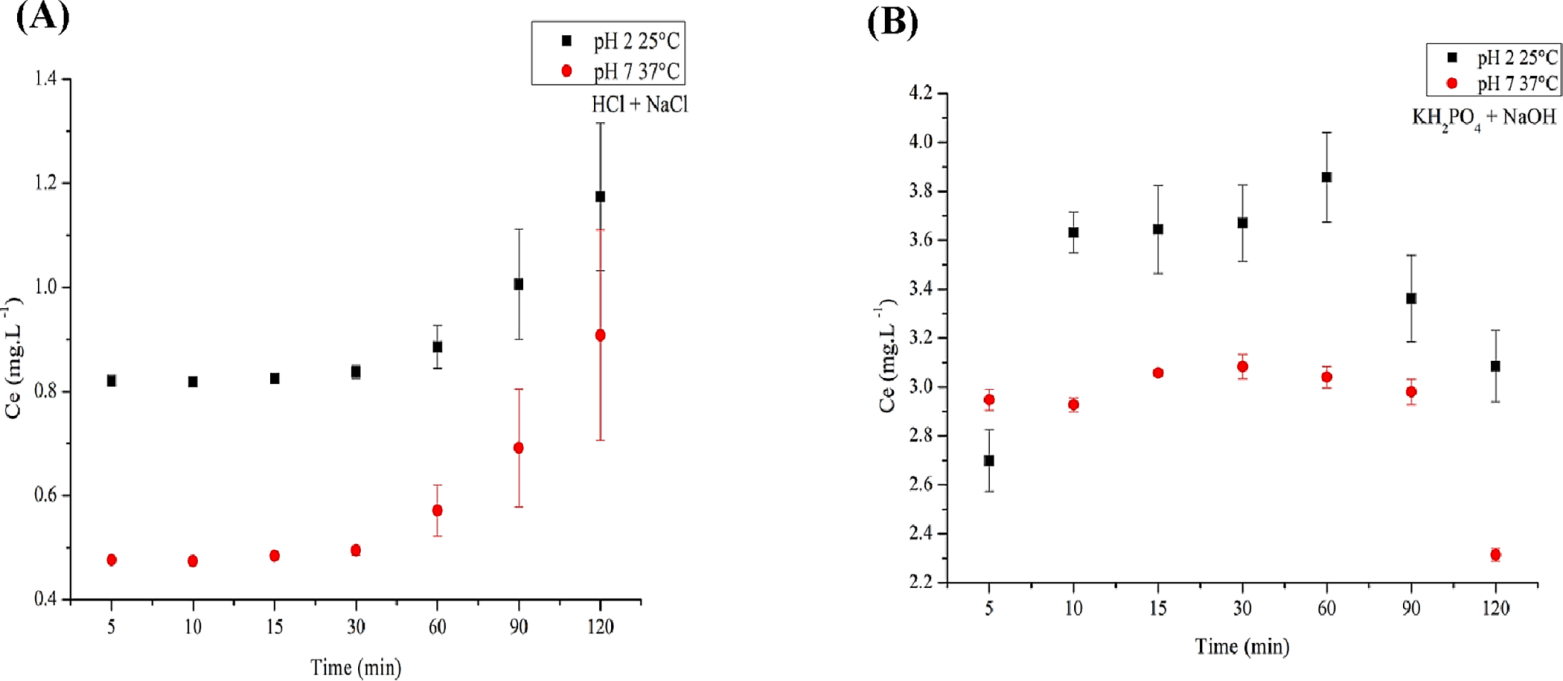
Simulated release profile of tartrazine dye adsorbed on BSY in gastric (A) and intestinal (B) environments. Experimental conditions: Samples containing 50 mL of tartrazine dye (10 mg·L^–1^) and 0.1 g of dry BSY were prepared and incubated at pH 2 (25 °C) and pH 7 (37 °C) for 2 h. After centrifugation (5000*g*, 10 min), the precipitate was transferred to buffer solutions at pH 2 or 7 (HCl or KH_2_PO_4_) and incubated at 37 °C under agitation. Samples of 3 mL were collected at 5, 10, 20, 30, 40, 50, 60, 70, 80, 90, 100, and 120 min and analyzed at 425 nm to determine the release of the dye from the BSY.

In the intestinal simulation (KH_2_PO_4_ + NaOH), dye release fluctuated at pH 2 and 25 °C but shifted when the temperature increased to 37 °C and the pH transitioned to neutral. This change weakened the interactions between the dye and BSY. In contrast, at pH 7 and 37 °C, dye release remained stable, resulting in less pigment dispersion.

Comparing the regions of the gastrointestinal tract, it was observed that BSY at pH 7 and 37 °C showed less release of tartrazine over time. This suggests a potential reduction in the adverse effects associated with lower levels of tartrazine in the body. However, future tests that more comprehensively simulate the gastrointestinal tract, accounting for the entire digestive and enzymatic process, will provide more accurate confirmation of this result.

Previous studies, which more accurately simulated gastrointestinal conditions and observed yeast digestion, such as those by Laurenti^54^ and Kil,^55^ indicated that fresh yeast (first use) remained intact under these conditions. Additionally, Rosa^47^ demonstrated that 94% of the cells remained viable after in vitro digestion, suggesting that fresh yeast can effectively reach the lower intestinal tract.

From a health perspective, this efficient retention of tartrazine dye is essential to reduce its release into the body and minimize adverse effects resulting from its degradation, which generates aromatic amines (anilines). In this context, inactivated yeast subjected to neutral pH and body temperature has been demonstrated to be effective in adsorbing and retaining the dye, representing a safe alternative to reduce systemic exposure to tartrazine and its potential negative impacts.^56–58^

## 4 Conclusions

This study demonstrated that inactivated *S. cerevisiae* (BSY), a byproduct of the brewing industry, is an effective biosorbent for the synthetic food dye tartrazine, exhibiting strong adsorption capacity and promising physicochemical interactions. The adsorption process was most efficient under acidic conditions (pH 2) and at 25 °C, where the yeast surface displayed greater positive charge, enhancing electrostatic interactions with the anionic dye. FTIR and TGA analyses confirmed the presence of key functional groups (hydroxyl, amine, and carboxyl) involved in hydrogen bonding, electrostatic interactions, and π–π interactions with the dye. Furthermore, these analyses revealed that both the dye and the biomass presented increased thermal stability after adsorption. SEM images provided additional support by highlighting morphological changes and dye deposition on the yeast surface, with pH influencing these effects.

Notably, although pH 2 was optimal for adsorption, simulated gastrointestinal experiments showed that pH 7 at 37 °C was more favorable for retaining the dye bound to the biomass, resulting in lower dye release. This indicates the potential of the BSY–tartrazine complex to act as a stabilizing matrix, potentially reducing the bioavailability of synthetic dyes in the human digestive system.

The findings of this work advance the use of inactivated BSY not only as a biosorbent but also as a functional ingredient capable of stabilizing colorants under physiological conditions. This opens new perspectives for the development of safer food formulations and for the valorization of industrial byproducts, promoting sustainable practices aligned with the circular economy. Future studies may explore regeneration and reuse cycles, in vivo validation, and integration of BSY–dye complexes into composite delivery systems for food and nutraceutical applications.

## Supplementary Material



## Notes

Standard curves of the dye tartrazine yellow at pHs 2 and 7: tartrazine solubility standard curve at pH 2 (Figure S1) and standard solubility curve for tartrazine at pH 7 (Figure S2) (PDF)
