# Supplementary material for: Brewer’s Spent Yeast as a Biosorbent for the Synthetic Dye Tartrazine Yellow
Source: ACS Omega. 2026 Feb 4;11(6):9022–37. doi: 10.1021/acsomega.5c05650 (PMC12917536; doi:10.1021/acsomega.5c05650)
Supplement: Supplementary file 1 [file ao5c05650_si_001.pdf]

Brewer's spent yeast as a biosorbent for the synthetic dye tartrazine yellow

Louise N. N. Lourenço[1], Ivaldo Itabaiana Jr[1] and Ailton C. Lemes[1]

[1]Department of Biochemical Engineering, School of Chemistry, Federal University of Rio de Janeiro, Rio de Janeiro 21941-909, Brazil.

SUPLEMENTARY MATERIAL

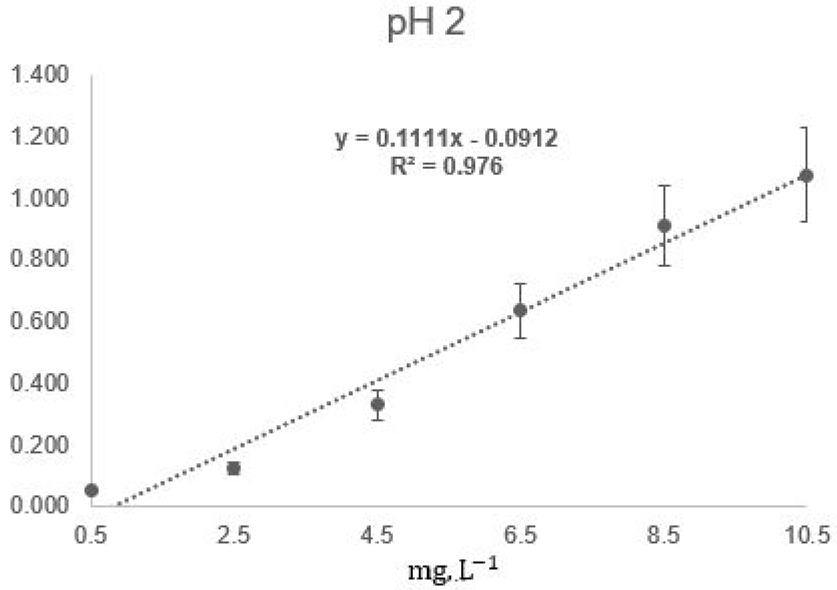

Figure S1: Standard curve tartrazine solubility pH 2.

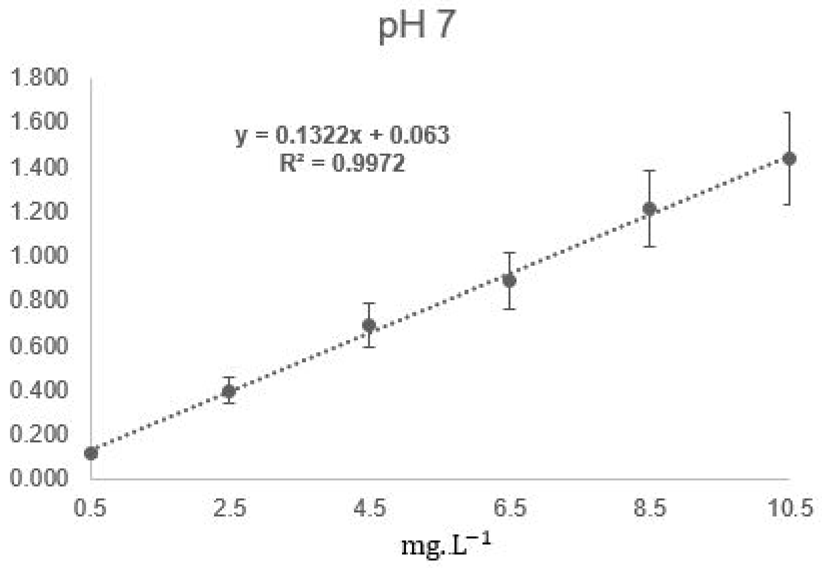

Figure S2: Standard curve tartrazine solubility pH 7.
